# Differential splicing using whole-transcript microarrays

**DOI:** 10.1186/1471-2105-10-156

**Published:** 2009-05-22

**Authors:** Mark D Robinson, Terence P Speed

**Affiliations:** 1 Department of Medical Biology, The University of Melbourne, Parkville, Victoria 3010, Australia; 2 Cancer Research Program, Garvan Institute of Medical Research, Darlinghurst, NSW 2010, Australia; 3 Bioinformatics Division, Walter and Eliza Hall Institute of Medical Research, Parkville, Victoria 3050, Australia

## Abstract

**Background:**

The latest generation of Affymetrix microarrays are designed to interrogate expression over the entire length of every locus, thus giving the opportunity to study alternative splicing genome-wide. The Exon 1.0 ST (sense target) platform, with versions for Human, Mouse and Rat, is designed primarily to probe every known or predicted exon. The smaller Gene 1.0 ST array is designed as an expression microarray but still interrogates expression with probes along the full length of each well-characterized transcript. We explore the possibility of using the Gene 1.0 ST platform to identify differential splicing events.

**Results:**

We propose a strategy to score differential splicing by using the auxiliary information from fitting the statistical model, RMA (robust multichip analysis). RMA partitions the probe-level data into probe effects and expression levels, operating robustly so that if a small number of probes behave differently than the rest, they are downweighted in the fitting step. We argue that adjacent poorly fitting probes for a given sample can be evidence of *differential *splicing and have designed a statistic to search for this behaviour. Using a public tissue panel dataset, we show many examples of tissue-specific alternative splicing. Furthermore, we show that evidence for putative alternative splicing has a strong correspondence between the Gene 1.0 ST and Exon 1.0 ST platforms.

**Conclusion:**

We propose a new approach, FIRMAGene, to search for differentially spliced genes using the Gene 1.0 ST platform. Such an analysis complements the search for differential expression. We validate the method by illustrating several known examples and we note some of the challenges in interpreting the probe-level data.

Software implementing our methods is freely available as an R package.

## Background

### Alternative splicing

Alternative splicing is the ubiquitous phenomenon where the same genetic locus can transcribe multiple messenger RNAs (mRNAs), by splicing out different subsets of intronic regions from a common pre-mRNA product. Splice variants of a gene can be functionally distinct and generate considerable proteomic diversity. Despite early estimates of near 50% [1], it is now thought that greater than 90% of all human genes exhibit alternative splicing [2,3], accounting for much of the complexity of metazoan organisms. Alternative splicing is known to be prominent in many important physiological processes, such as cell differentiation, apoptosis and development, and is especially prevalent in the nervous system [4-6]. Mis-regulation or mutations that affect the splicing mechanism can result in disease, including cancer [7]. It is no surprise then that alternative splice variants have been observed in a tissue-specific or cancer-specific manner.

Until recently, predicting alternative splice events usually involved the comparison of expressed sequence tags (ESTs) across several libraries. For example, algorithms that compare EST abundance across human tissues deduced many tissue-specific isoforms [8,9]. Recently, DNA microarrays have been successfully utilized to explore alternative splicing, finding many genes with known and putative tissue-specific isoforms [1,10,11].

In this study, we propose a statistical method of scoring differential splicing for the Gene 1.0 ST (hereafter referred to as **Gene**) array data, which is the latest generation of Affymetrix genome-wide expression profiling chips. Note that the aim of this work is not to suggest the **Gene **platform as a replacement for the Exon 1.0 ST (referred to as **Exon**) array. The considerations of cost, probe coverage and protocol (e.g. amount of RNA needed) will ultimately guide this decision for experimenters. We expect the **Gene **platform will be used regularly for expression profiling studies and here, we describe the potential to identify differential splicing at no additional experimental cost. We are simply providing a additional data analysis-based avenue of interrogation. Here, our motivation is to outline the possibilities and limitations of using the **Gene **array for the detection of differential splicing, not to rigourously compare and contrast the platforms.

Both the **Gene **and **Exon **arrays interrogate well-annotated exonic content. Perhaps not surprising given the two platforms share a large number of probes, we have discovered that many of the patterns observed in **Exon **data are also observed in **Gene **data. In addition, we note some of the challenges and ambiguities of analyzing whole transcript microarray data in the context of alternative splicing.

We have shown previously that **Gene **has similar performance to **Exon **and a previous generation of Affymetrix chips, in various respects in the context of gene expression [12]. There is certainly value in having an expression platform that can additionally deduce alternative splice forms. **Exon **does this [12,13,19]. We show here that **Gene **has potential to do so as well, if we are willing to interrogate only well-annotated content and have reduced coverage for some transcripts.

### Differential splicing

It is worth noting at the outset that microarrays, in general, will not be able to detect alternative splicing, *per se*. For example, if an exon is spliced out of all tissues or samples in the study, there is no ability to detect it as alternative splicing. So, the focus of the methodology presented here and other related methods is on detecting *differential *splicing, or more generally, the differential expression of alternative isoforms.

### Affymetrix array design

**Figure 1:**
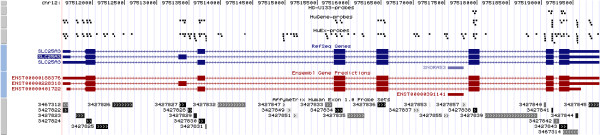
**UCSC browser view of solute carrier family 25, member 3 (SLC25A3).** Custom tracks have been added for the locations of the 25-mer probes for the Affymetrix **Gene**, **Exon **and HG-U133 human expression arrays, relative to the locations of exons for RefSeq or Ensembl gene builds. The **Exon ***probesets *are shown in black and grey in the lowermost track. Several probes are common to both the **Gene **and **Exon **platforms.

Figure 1 shows a UCSC browser view [14] of the locations of **Exon **probes and probesets and **Gene **probes for a single human gene, *SLC25A3 *(solute carrier family 25, member 3). As is standard with Affymetrix design, all probes are 25 base pairs, however, on the newer generation of chips, there is no mismatch probe for every perfect match probe. The **Exon **probesets, one for each probe selection region (PSR), are shown in black for well-annotated exons and 2 shades of grey depending on the original prediction evidence. PSRs are defined by Affymetrix according to whether a particular region may act as an independent unit, based on several levels of annotation projected to the genome. The array design for **Exon **aims to have 4 probes per PSR whereas the **Gene **array has approximately 25 probes per transcript cluster [12].

The **Gene **platform shares a large number of its probes (approximately 65%) with the **Exon **array, but also includes a significant number of probes unique to the platform. In terms of differential splice detection, the coverage by either platform is locus-specific. The ability to detect differential isoform usage will depend not only on the number of probes covering the region, but the nature of the splicing, the degree of differential usage and the performance of the probes near to the event. This could also mean there is a bias in the ability to detect differential splicing with **Gene **through genes having fewer rather than more exons. In general, genes with fewer than 5 or 6 exons will have more probes per exon on the **Gene **array. We have not studied this possible bias in any detail. Instead, we focus on determining differential splicing predictions based on the available data with the current **Gene **design.

### RMA decomposition

**Figure 2:**
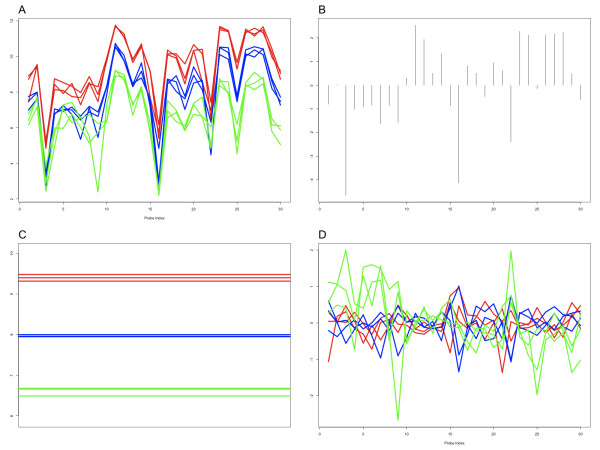
**RMA decomposition of probe-level Affymetrix data**. Panel A shows the background-adjusted and normalized probe-level data for *PRRX1*, from the Affymetrix mixture dataset (see Methods). The probes are displayed in the order which they map to the human genome (not to scale), and lines join all probe intensities of the same sample. *PRRX1 *is expressed significantly higher in heart tissue compared to brain. Three replicates of pure heart tissue are shown as red lines; green lines represent pure brain tissue replicates and the blue lines represent a mixture of 75% brain tissue and 25% heart tissue. Panel B shows the estimated relative probe effects. Panel C shows the chip effects (i.e. summarized expression levels) and Panel D shows residuals, using the same colour scheme.

After background adjustment and normalization, one of the commonly used methods for summarizing probe-level Affymetrix data into expression levels is robust multichip analysis (RMA) [15]. The approach accounts for relative probe-specific effects according to the following model:

(1)

where *Y*_*ij *_are the log_2 _background adjusted and normalized intensities for probe *j *from sample *i*, *α*_*i *_are the *chip *effects (*i *= 1, ..., *N*) and *β*_*j *_are the *probe *effects (*j *= 1, ..., *J*), given *N *samples and *J *probes and *ε*_*ij *_are the errors. For simplicity, a subscript for gene is suppressed here since all models are fit to genes one by one. The constraint  is imposed to make the probe effects relative and identifiable. Figure 2A illustrates probe-level data for a gene that is strongly differentially expressed between heart and brain across a full mixture of RNA samples (red – 100% heart, green – 100% brain, blue – mixture). The most striking observation of the probe-level data is the parallelism across all samples, largely due to the sequence-specific probe intensity effects. That is, because this gene is differentially expressed between brain and heart, each individual probe shows a relative change in abundance, even though the range of intensity for each probe may be quite different. RMA models this behaviour by estimating probe-specific effects (Figure 2B), leaving the relevant sample-specific features (chip effects, Figure 2C) for downstream analysis of expression. The residuals (Figure 2D), which are the differences between the observed intensities and that explained by the model, are random and centred around 0. The models are fitted robustly using iteratively reweighted least squares [16] so that individual observations do not have undue influence in the estimation of *α*_*i *_and *β*_*j*_.

**Figure 3:**
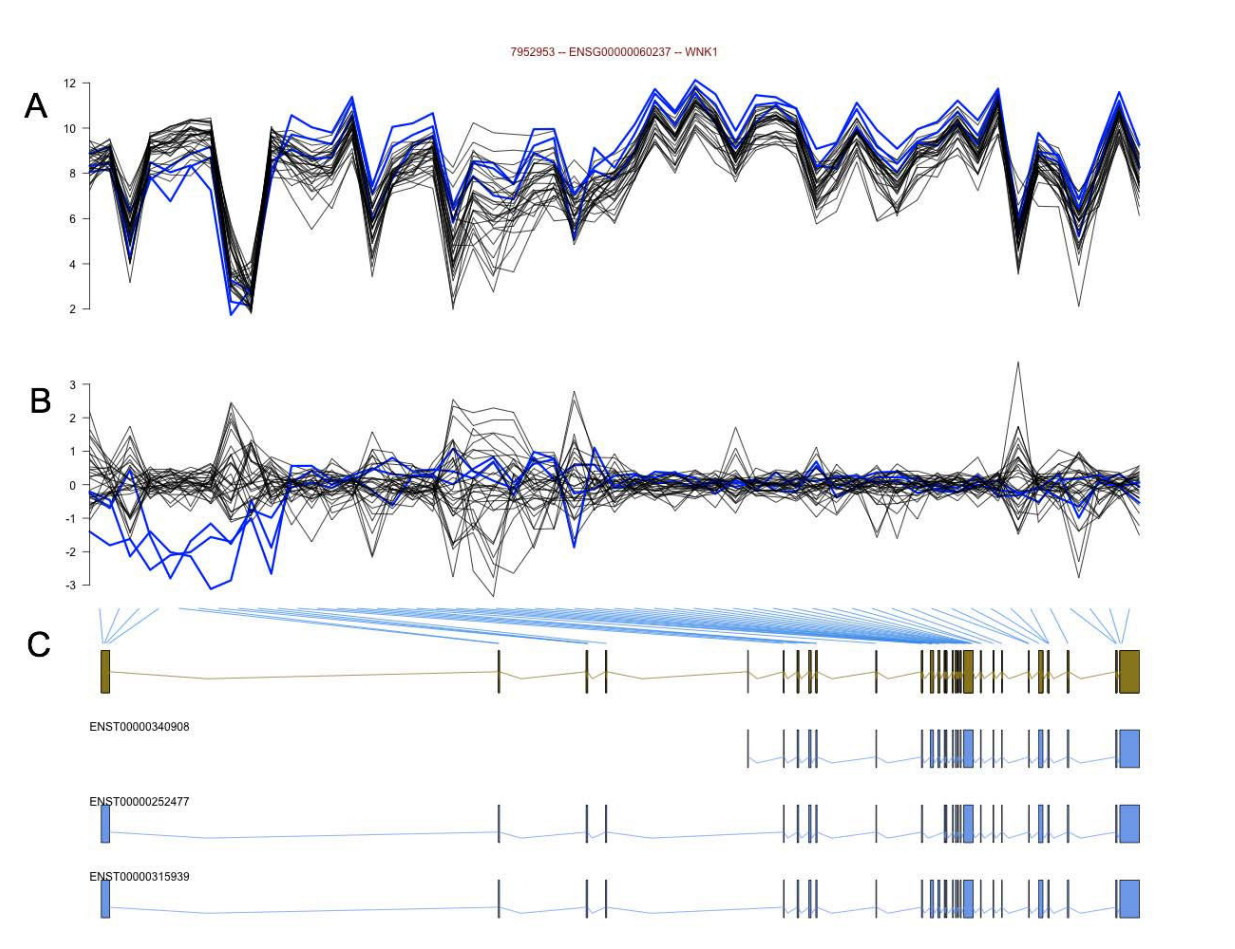
**Differential splicing of WNK1**. Panels A and B show the normalized data and residuals, respectively, of *WNK1 *for the Affymetrix tissue dataset (see Methods). The three replicates for human kidney tissue are shown as blue lines, and the remaining 10 tissues (30 samples) are shown with black lines. Panel C shows the set of exonic regions joined together in a *gene model *(green) and the three known Ensembl transcripts (blue). The blue lines linking Panels B and C illustrate the correspondence between probes and exons.

Next, we show that alternative splicing can be highlighted by focusing on the residuals. Take for example *WNK1 *(lysine deficient protein kinase 1), a gene known to express a kidney-specific isoform having a 5' region spliced out [8]. Figure 3 shows the probe-level data for *WNK1*, as well as the residuals after fitting the RMA model. Figure 3A illustrates quite clearly that several probes near the 5' region of the gene for *WNK1 *are expressed at noticeably lower levels in the kidney samples than in the remaining samples, as would be expected. Since the RMA model is fitted robustly, the 5' probes for the kidney samples, which depart from the parallelism we saw previously, are downweighted, and so have a relatively small influence on the overall estimation of chip and probe effects. However, for the determination of differential splicing, these observations in the 5' region of the gene are very much of interest. Figure 3B highlights a sequence of residuals that appear very different from the rest of the gene. We return to this observation in the next Section. Figure 3C shows the genomic context of the probe-level data and the known Ensembl trancripts for *WNK1*. The sequence of residuals showing the persistently low values suggest kidney-specific expression of the short transcript *ENST00000340908*, in agreement with the previously published result [8].

### Related Work

To the best of our knowledge, this paper is the first attempt at using the **Gene **platform to investigate alternative splicing. Several methods have been proposed for the differential splicing analysis of **Exon **data, including the Splicing Index (SI) [10], pattern-based correlation (PAC) [17], microarray analysis of different splicing (MADS) [18] and finding isoforms using the robust multichip average algorithm (FIRMA) [19]. The SI forms a score that represents the difference between the gene-level summary (as fitted by an RMA-like algorithm) and an exon-level summary, requiring two estimation steps. Effectively, the method estimates probe effects twice independently, one with all probes for the gene and another with only the probes for a probeset. We do not see SI as a feasible approach with **Gene **data, since even if we were to create probesets that represent exons, often very few probes will available and it will be difficult to get reliable estimates. The fewer probes per probeset will have a similar effect on applying FIRMA directly to **Gene **data. FIRMA fits the standard RMA model (as above) to all probes for a given gene and summarizes probeset-wise departures from the model through the residuals. With very few probes, the probeset summaries of residuals may not be precise. PAC, on the other hand is an *all-sample *approach that scores each probeset on whether it correlates with the rest of a gene, over all samples. Simulation studies for a modest number of chips (e.g. 20) show that FIRMA and SI generally outperform PAC [19]. MADS is a new approach for **Exon **data that combines several steps, including probe selection and compensation for sequence-specific cross-hybridization effects. Though it has not been applied to the **Gene **platform, it appears that since calculations are done at the probe level and combined together to make inferences about probesets, it may be possible to adapt the method.

### Differential splicing

#### Scoring persistence of residuals

The method presented in this paper differs from previous approaches in that we focus on identifying *genes *with possible alternative splice forms, instead of highlighting exons or probesets. This has a subtle statistical advantage in that the multiple testing penalty is considerably smaller.

As mentioned above, the residuals from the RMA model hold the key to finding differential splicing events. Instead of focusing on individual exons (and the organization of annotation that that requires), we score a persistent deviation from zero of adjacent residuals. The residuals are defined as:

(2)

where  and  are estimated using robust fits of Equation 1. In order to normalize across genes, we calculate standardized residuals  = *r*_*ij*_/*MAD*{*r*_*uv*_, *u *= 1, ..., *N*; *v *= 1, ..., *J*} where *MAD*(.) is defined as 1.4826 times the median absolute deviation from 0 over all residuals for that gene and all samples. In the absence of alternative splicing, standardized residuals will have approximately unit variance. FIRMA [19] takes advantage of the **Exon **array design, where each PSR has 4 probes and residuals can be summarized at the probeset level. If a particular PSR is differentially spliced, then it is expected that most if not all probes for the PSR would have a large-in-magnitude residual (i.e. not fit well by the RMA model). For the **Gene **array, we are not guaranteed 4 probes per exon and, depending on the probes designed for a particular transcript, may have very little power to detect single exons that are differentially spliced. Since the performance of the summary will be related to the number of observations used to calculate it, we consider an alternative procedure. We take the approach of finding a persistence of residuals that are away from zero and in the same direction, thus entirely avoiding the non-uniformity of probes per exon. We only require that the probes are put in genomic order for the calculations below. Several adjacent probes, interrogating exon regions that are adjacent in an mRNA product, that show the same departure from the model are evidence of potential differential splicing. For example, Figure 3 illustrates that probes 2–8 of *WNK1 *all have strongly negative residuals. Such an observation is unlikely to occur by chance.

One possibility to highlight such persistence of residuals is an extreme value of the absolute values of all partial sums of adjacent residuals. A natural statistic, inspired by the monitoring of nuclear material unaccounted for (MUF) [20], is the maximum absolute partial sum:

(3)

over the *J*(*J *+ 1)/2 possible consecutive sums of *J *probes. This calculation is repeated separately for each gene, giving a score for each gene and each sample. That is, this approach can be applied in the absence of replicates. However, if replicates are available, we recommend precomputing a probe-wise summarized residual  and use this in place of  in Equation 3, where *i*(*k*) is the index of replicate *k*.

The MUF statistic is very flexible. An extreme MUF statistic can result from a single probe if it is extreme enough. But, it can also highlight a subtle change that persists across any number of probes if the score is deemed to be extreme. Notice that the denominator of the partial sums is the square root of the number of data points. This ensures the variances (of the sum) are constant, thus putting all the partial sums on an even footing.

As the number of probes increases, there are more partial sums to consider, making the distribution of maximum order statistics more likely to take on more extreme values. To alleviate this, we repeatedly sample *J *probes from the empirical distribution of all standardized residuals and calculated the MUF score, giving a null distribution of MUF scores for *J *probes. A false discovery rate can be calculated for the discoveries above a given quantile of the null distributions.

We call this approach FIRMAGene, since it is only a small modification to FIRMA [19], in terms of operating on residuals from an RMA fit, but is applied to the Affymetrix Gene 1.0 ST platform and scores differential splicing at the gene level instead of the probeset level.

## Results

### Validation of using the Gene platform for splicing

**Figure 4:**
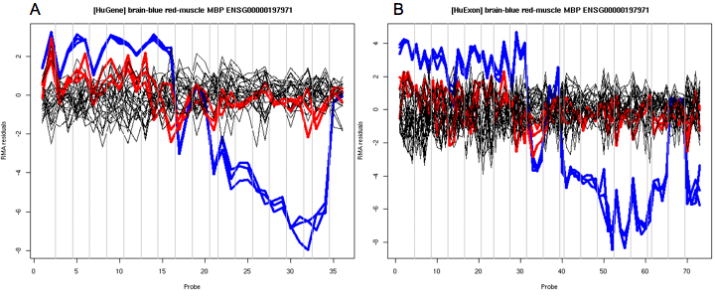
**Normalized probe-level data and RMA residuals for MBP**. Panels A and B show the residuals for **Gene **and **Exon **for RMA fits, respectively. There are 36 probes for **Gene **and 72 probes for **Exon**. Both panels show 33 lines, one for each hybridization (11 tissues with 3 biological replicates each). The brain and muscle replicates are shown blue and red lines, respectively.

**Figure 5:**
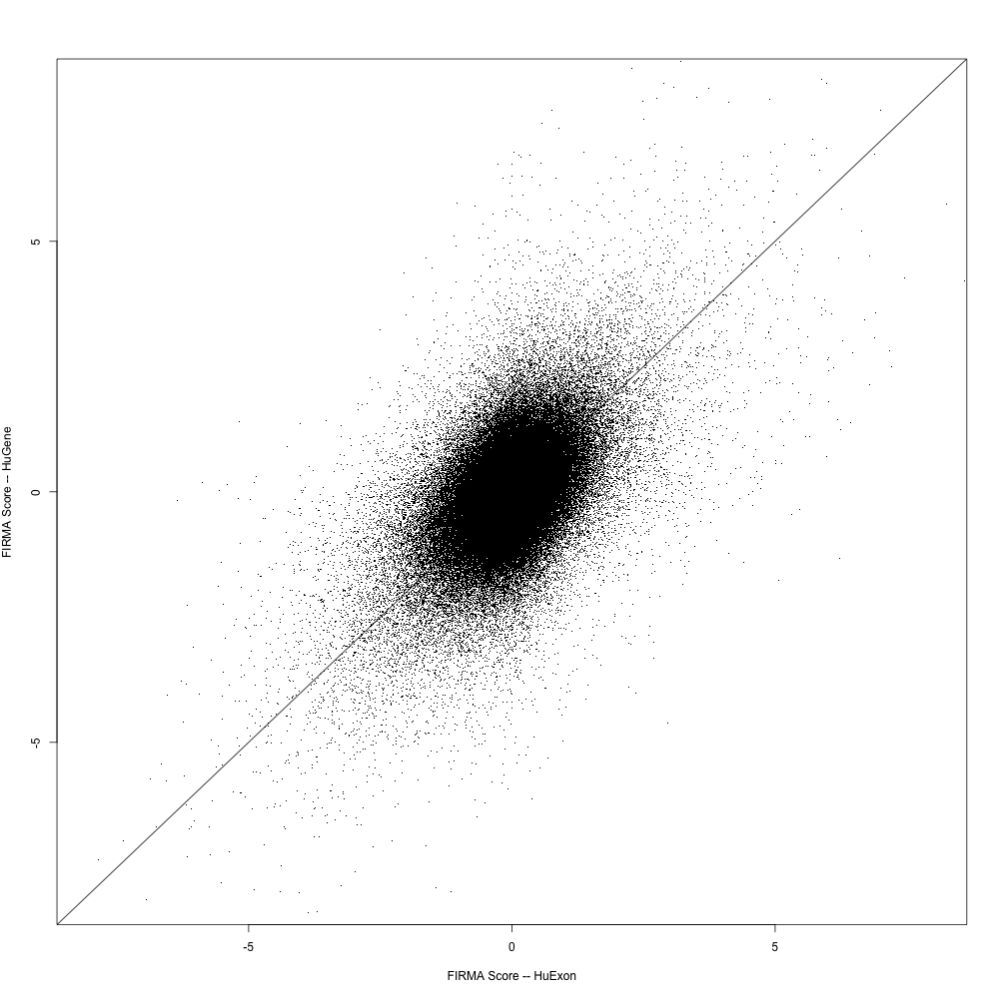
**A comparison of FIRMA scores for Gene and Exon platforms**. Each point in the scatter plot represents an **Exon **probeset that has been matched to probes on the **Gene **array. The X-axis gives the averaged (over brain replicates) FIRMA score for **Exon **data. The Y-axis gives the average FIRMA score for the corresponding **Gene **samples.

We first validate the approach of using the **Gene **platform for differential splice detection by comparing the residuals for a gene known to express a vastly different isoform in human brain [21], using the publicly available data of the same tissue RNA hybridized to both the **Gene **and **Exon **platforms. Figure 4 shows residuals plots for *MBP *(myelin basic protein) and highlights a very distinct pattern in the brain samples. This pattern is observed almost identically from the 36 probes represented on the **Gene **array or 72 probes from the **Exon **array. The exact splicing mechanism is not as apparent as in the previous example (WNK1, Figure 3), but it is straightforward to put the probe-level data in the context of known genome annotation. For a genome-wide comparison, we matched the probes from the **Gene **array to the Affymetrix-defined probesets of the **Exon **array, allowing us to run FIRMA on the **Gene **platform. Note that we are not advocating the use of FIRMA for the **Gene **platform, although we do highlight that it can be done and allows us to make the comparison. See Methods for further description of procedures used to construct the annotation. FIRMA scores are calculated for **Gene **and **Exon **data, generating a table of scores by probeset and sample, one for each platform. Note that the summaries for the two platforms are often from different numbers of probes and therefore have different precision. Table 1 give a cross-tabulation of the numbers of probes for both platforms amongst matched probesets. The **Exon **array most often has 4 probes per probeset, whereas the **Gene **platform most often has 1 or 2 probes. In some cases (e.g. genes with few exons), **Gene **will have more than 4 probes. Taking the average of FIRMA scores over the 3 brain replicates, Figure 5 illustrates convincing genome-wide evidence that extreme residuals observed on the **Exon **array are also observed on the **Gene **array (correlation *r *= 0.53 over more than 230,000 **Exon **probesets). This is especially promising considering the majority of summarized sets of residuals will be centred close to 0. Shown in Figure 5 are summaries for the brain replicates from each platform, since brain tissue is expected to exhibit more alternative splicing than most other tissues.

**Table 1:** Number of probes per matched probeset. After taking a subset of common probesets, the numbers of probes for each matched Exon probe selection region are given.

No. of **Gene **Probes					
No. of **Exon **Probes	1	2	3	4	5
1	6802	0	0	0	0
2	2654	4191	6	1	1
3	2794	2788	1155	11	7
4	87092	83621	24219	12301	4019

**Figure 6:**
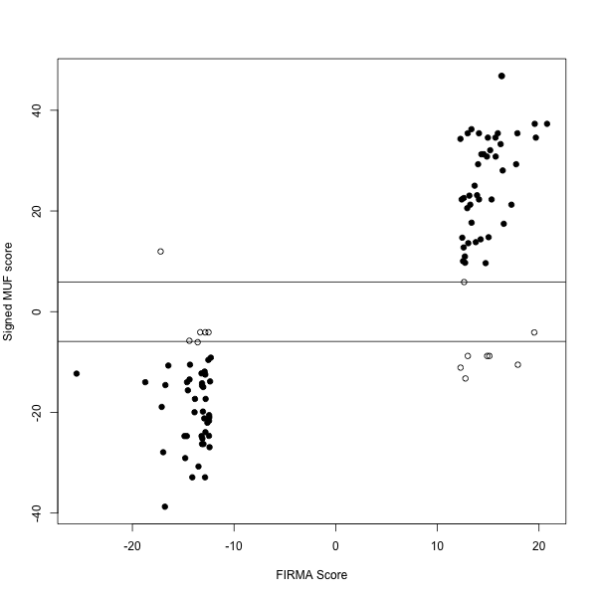
**FIRMAGene scores for the top 100 FIRMA scores for the Affymetrix tissue panel dataset**. The X-axis gives the FIRMA score (calculated on the Affymetrix **Exon **tissue panel dataset) for the top 100 probesets with 4 probes. The Y-axis gives the signed MUF scores (calculated on the Affymetrix **Gene **tissue panel dataset) for the corresponding genes. Circles which are filled in correspond to MUF scores that are more extreme than the 95^th ^percentile of the permutation-based null distribution.

Next, we were interested to determine whether **Gene **data is able to detect a significant proportion of the differential splicing events that FIRMA detects on **Exon **data. The tissue panel dataset, where the same source of RNA is hybridized to both platforms, is an ideal test set for this comparison. We applied FIRMA to the **Exon **data and FIRMAGene to the **Gene **data. We compared the top 100 probeset-tissue scores from FIRMA to the corresponding gene-tissue scores from FIRMAGene, as shown in Figure 6. The vast majority of the MUF statistics are large in magnitude, suggesting that **Gene **platform is quite capable of detecting similar differential splicing events. In fact, 86 of the 100 corresponding gene-tissue scores have MUF statistics more extreme than the 95^th ^percentile of their null distribution. One the other hand, because FIRMA gives (sub-)exon-level and FIRMAGene gives gene-level statistics, there may be some cases where the scores do not correspond. For example, 4 of the 14 MUF statistics that are not extreme have no **Gene **probes represented in the region where the **Exon **probes are. Furthermore, since the MUF score is an extreme value statistic, there may be set of probes within the gene that are more extreme in the opposite direction, as shown in 8 of the 14 non-extreme MUF scores. Overall, this analysis suggests that the **Gene **platform will be quite promising for the analysis of differential splicing.

### Tissue panel dataset

The publicly available 11 tissue panel dataset, where the same human tissue RNA was run on both the **Gene **and **Exon **platforms in a single laboratory by Affymetrix, provides an ideal testing ground for the methodology and for illustrating of some of the features of whole-transcript microarray data. Although there are many individual examples in the literature, there is no readily available positive control set of tissue-specific alternative splice events that can be used for benchmarking. However, tables of EST-based predictions exist. A rigorous comparison of EST predictions and microarray analysis of alternative splicing events is beyond the scope of this study. Instead, we calculate scores genome-wide (using **Gene **data) across the 11 tissues and show that many of the top ranking scores have been observed previously to either have tissue-specific variants or tissue-specific expression patterns.

**Figure 7:**
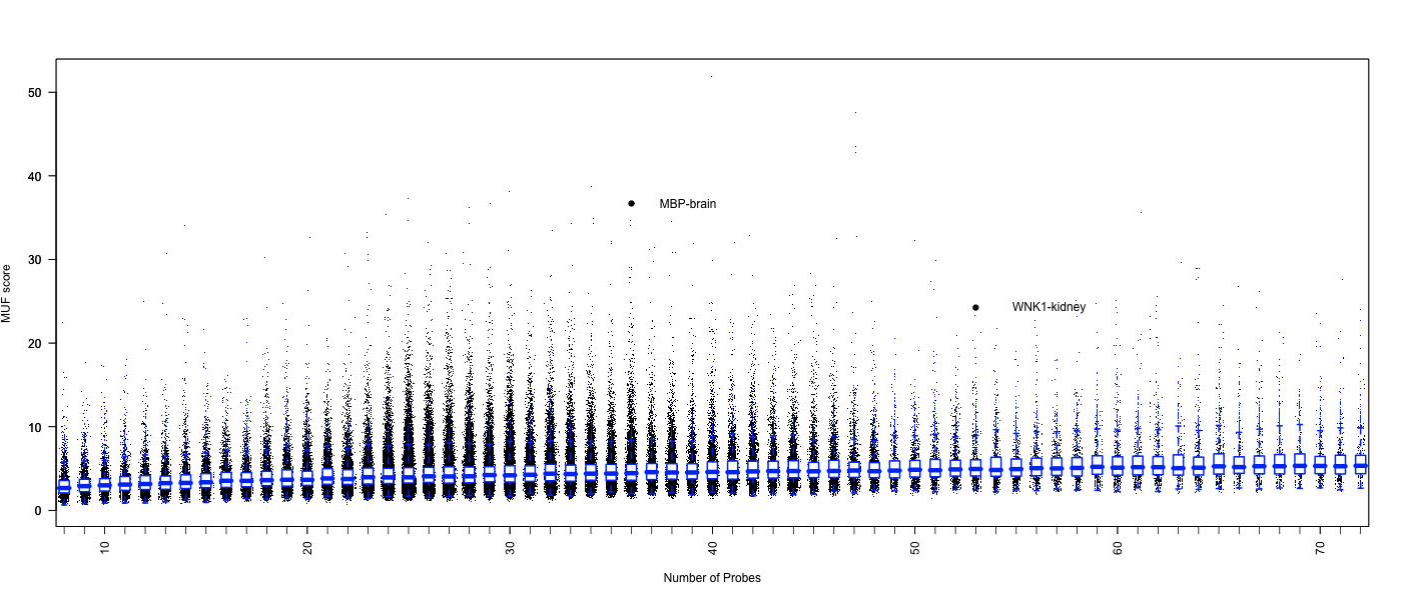
**FIRMAGene scoring for the Affymetrix tissue panel dataset**. Each point in the plot gives a FIRMAGene score for a tissue-gene combination. The X-axis gives the number of probes and Y-axis gives the raw MUF score. Jitter is added in the X dimension. The simulated null distribution is shown as blue boxplots. The scores for gene-tissue combinations shown in Figures 3 and 4 are highlighted.

Figure 7 shows the genome-wide scores, stratified by the number of probes for each gene. The plot shows only the genes that have between 10 and 70 probes (nearly 95% of the genes on the array). Because genes with more probes have more partial sums to consider, the maximum gently increases with the number of probes per gene. The two examples shown earlier, *WNK1 *for kidney tissue and *MBP *for brain tissue, have high scores, as highlighted. Table 2 shows the top scoring gene-tissue combinations. Of the top 20 gene-tissues scores, many of them have previous evidence of tissue-specific behaviour. Plots of the normalized data and residuals can be found in Additional file 1, in addition to a list of publications corroborating the tissue-specific evidence.

**Table 2:** Top scoring tissue-specific differential splicing candidates.

ID	Sample	Score*	Symbol
7922737	Testis	24.76	C1orf14
8086077	Brain	21.84	CLASP2
8086842	Brain	20.99	MAP4
7957746	SkMus	19.76	SLC25A3
7957746	Heart	19.41	SLC25A3
8165653	Heart	18.72	--
8166876	Testis	18.29	DDX3X
8064191	Brain	18.08	TPD52L2
8007188	Brain	18.01	CNP
7922627	Kidney	18.01	NPHS2
8170215	Liver	17.93	F9
8100458	Testis	17.84	PDCL2
7962194	Testis	17.78	LOC440093
7940971	Testis	17.56	KCNK4
8176419	Testis	17.18	TSPY2
8155203	Brain	16.97	CLTA
8170390	Brain	16.84	--
8023889	Brain	16.79	MBP
8176544	Testis	16.64	TSPY1
8024194	Testis	16.32	GPX4

The Affymetrix Human Gene 1.0 ST identifiers and gene symbols are given for the top 20 tissue-gene combinations.

*See Methods.

Some tissues have considerably more differential splice detections. For example, of the top 1000 gene-tissue scores (see Additional file 2), the top three tissues are testis (295), brain (258) and liver (116). This corresponds with previous EST studies where brain, liver and testis have the highest percentage of alternatively spliced genes [22].

## Conclusion and Discussion

We have proposed a novel scoring method called FIRMAGene based on decomposing probe-level microarray data with a linear model. The major motivation for this work is to provide an extra investigation, in addition to differential expression analysis, thus giving researchers added value from their collected data. The design of the latest generation of Affymetrix expression array facilitates this. Using a public tissue panel dataset, we show the method highlights many previously known and potentially new differential tissue-specific splice events and shows strong correspondence with the **Exon **array over the same RNA samples. The strategy we propose can be applied directly to the Affymetrix Human, Mouse and Rat Gene 1.0 ST platforms, or any other whole-transcript platform that exhibits probe-specific effects. Although we have not investigated thoroughly, FIRMAGene may be useful for the **Exon **array. It comes at some additional computational cost, since there are more probes (and therefore even more partial sums), but it may be better able to highlight smaller, but persistent, changes in adjacent probesets. The procedure can operate in a single sample mode or can make use of replicates. Technical or biological replicates can be used, although significant detections from the latter will give more generalizable results. One subtle difference that FIRMAGene takes advantage of, is the fact that scoring by gene instead of by exon results in a much smaller penalty for multiple testing.

The **Gene **platform will be used in various profiling studies and this work simply provides an additional analysis that will be of interest. The approach is not without limitations. The **Gene **platform only covers well-annotated exons, whereas the **Exon **platform covers a considerable amount of additional content, based on either EST evidence or computational predictions. However, having no features representing predicted exonic content has some advantages. For example, in the analysis of **Exon **data, it is not always clear whether to include all probesets (for well-characterized *and *predicted exons together) in the RMA model fit. The MADS approach uses a computational probe selection for this [18]. In many cases, the probes for content with weak evidence are not used for the primary analysis [19]. Since the well-characterized exonic content on the **Exon **array only represents approximately 20% of all features, the selection of probesets to include may have a large impact on the differential splice detections. In addition, for short genes, the **Gene **platform will generally have more probes than the **Exon **array, giving potentially higher power to detect new variants.

Since we are scoring a gene over all partial sums of probes, the MUF score is very flexible. It simultaneously searches for extreme residuals over any number of adjacent probes, including a single probe if it is extreme enough. There are variations of the MUF score that may be worth pursuing for a more refined mapping of differential splice events. For example, it is generally unreasonable that all residuals for a single sample will be non-zero. It may suffice to consider only partial sums of length less than ⌈*J*/2⌉, for example. Another variation would be to target specific patterns. For example, *SLC25A3 *(solute carrier family 25, member 3) has a very distinct mutually exclusive differential splicing pattern (see Additional file 1). If this or other distinctive patterns were of particular interest, the scoring of adjacent residuals could be tailored towards it.

It is difficult to know in what experimental circumstances the **Gene **platform and a procedure such as FIRMAGene will be most successful. We have shown FIRMAGene can be useful in a panel of tissues, where in general the majority of samples exhibit the same probe-level pattern and only a small number of samples differ. We expect the procedure will be useful even in a balanced two group comparison, where differential isoform usage would still present as a persistent departure from the linear model. However, there may be limitations in the robust fitting for probe effects in cases where the probe intensities are split into two distinct groups. One possible option would be to use existing **Gene **data (e.g. from a public source), in order to stabilize the probe effect determination. We have not investigated this thoroughly. As mentioned above, microarrays are only able to detect differential splicing, so in order to detect such events, there needs to be enough variation amongst the samples for a pattern to stick out. Depending on the strength of the difference and the number of probes represented on the array for the alternative spicing event (which can vary from gene to gene), a large sample size may be required.

Identifying departures through residuals from the RMA model will not always be perfect. Some departures from the RMA linear model may not be alternative splicing at all. In some cases, large residuals may be a result of cross-hybridizing probes, or through probes that have a different range of intensity, or are induced through, for example, an exon that is not expressed in any of the samples in combination with strong differential expression. It may be possible to compensate for cross-hybridization, as demonstrated recently (see [23,24]). With relevance to studies involving human populations, it has been recently shown that single nucleotide polymorphisms can significantly affect probe-level **Exon **data [25]. In addition, a resource has now been created to track **Exon **probes that may be affected [26]. Individual probe performance aside, we argue that most of the detected examples are biologically meaningful and these problems are not isolated to FIRMAGene and represent the challenging nature of designing methodology that operates over a range of probe behaviours. Other procedures, such as SI, MADS or PAC if they were to be adapted to the **Gene **platform, would need to effectively deal with these same challenges.

There are a number of other issues that we are aware of, but are beyond the scope of this investigation. For example, in some cases, the probes for a gene are overlapping. This may induce a correlation between residuals of neighbouring probes. The current model assumes independence for all probes and makes no compensation for this.

As evidenced by the top ranked genes, our current scoring scheme seems reasonable and does highlight interesting cases. Despite the limitations mentioned above, this research highlights an additional avenue of investigation beyond differential expression that is freely available at a minimal additional computational cost.

## Methods

### Datasets

The mixture dataset used for illustration of RMA (Figure 2) and the tissue panel datasets (**Gene **and **Exon**) were run by Affymetrix and made publicly available (see ). Briefly, the mixture dataset comprises 33 total samples, 3 technical replicates each of 11 separate mixtures. The tissue panel datasets use the same RNA on both the **Gene **and **Exon **platforms. Again, there are 33 total samples representing 3 biological replicates of each of the following human tissues: brain, thyroid, breast, pancreas, prostate, heart, skeletal muscle, kidney, testis, spleen and liver.

### Data processing

All data processing has been performed in the open source statistical package R[27] and the methods implemented in this paper are available from the authors as an R package, operating on objects created using the aroma.affymetrix package [28]. *Chip definition files *(CDFs) have been created for both arrays, based on library files and annotation made available from Affymetrix, using the Bioconductor [29] affxparser package. To facilitate alternative splicing analysis, probe collections are organized in a gene-centric fashion, so that probes from all known isoforms for a gene can be analyzed by a single framework (i.e. fit with the RMA model). For the **Exon **platform, we are used *core *probesets only. For the **Gene **array, the coordinates of the probes are matched to the **Exon **probeset coordinates, so that summaries for the same regions can be compared. Some probes for the **Gene **array, however, fall outside the region of **Exon**'s PSR. These are still kept within the **Gene **probe collection, but not used for the comparison.

Running FIRMAGene consists of the following steps: 1) fit the RMA probe-level model robustly for each gene, 2) standardize the residuals by dividing by the gene-wise MAD and summarize over residuals if replicates are used, 3) calculate the maximum MUF score for each sample, 4) given the number of probes for a gene, sample a large number of vectors of residuals (from the empirical distribution of all residuals) of same length, calculate the MUF score on each one to generate the null distribution, 5) at a given cutoff, calculate the false discovery rate. An example R script for running these steps on the tissue dataset is provided in Additional file 3.

The score represented in Table 2 compares the tissue-gene score to the mean and standard deviation of the permutation-based null distribution (subtract mean, divide by standard deviation).

## Authors' contributions

MDR conceived the original idea, analyzed the data, implemented the software and wrote the paper. TPS refined the statistical analysis and directed the project.

## Supplementary Material

Additional file 1 **Plots and corroborating evidence for the top 20 gene-tissue scores**. Probe-level data and residuals for the top 20 gene-tissue scores, from applying FIRMAGene to the Affymetrix tissue panel dataset. Additionally, links to various corroborating evidence of tissue-specific splicing or expression. Click here for file

Additional file 2 **Top 1000 Gene-tissue scores for the tissue panel dataset**. Table giving the probeset identifier, tissue sample, FIRMAGene score and gene symbol, after applying FIRMAGene to the Affymetrix tissue panel dataset. Click here for file

Additional file 3 **Example **R** script for FIRMAGene (R)**. Source code example to run FIRMAGene on the Affymetrix tissue panel dataset. Click here for file
