# Supplementary material for: Differential splicing using whole-transcript microarrays
Source: BMC Bioinformatics. 2009 May 22;10:156. doi: 10.1186/1471-2105-10-156 (PMC2703633; doi:10.1186/1471-2105-10-156)
Supplement: Additional file 1 — Plots and corroborating evidence for the top 20 gene-tissue scores. Probe-level data and residuals for the top 20 gene-tissue scores, from applying FIRMAGene to the Affymetrix tissue panel dataset. Additionally, links to various corroborating evidence of tissue-specific splicing or expression. [file 1471-2105-10-156-S1.pdf]

7922737 -- ENSG00000157060 -- C1orf14 -- blue=Testis

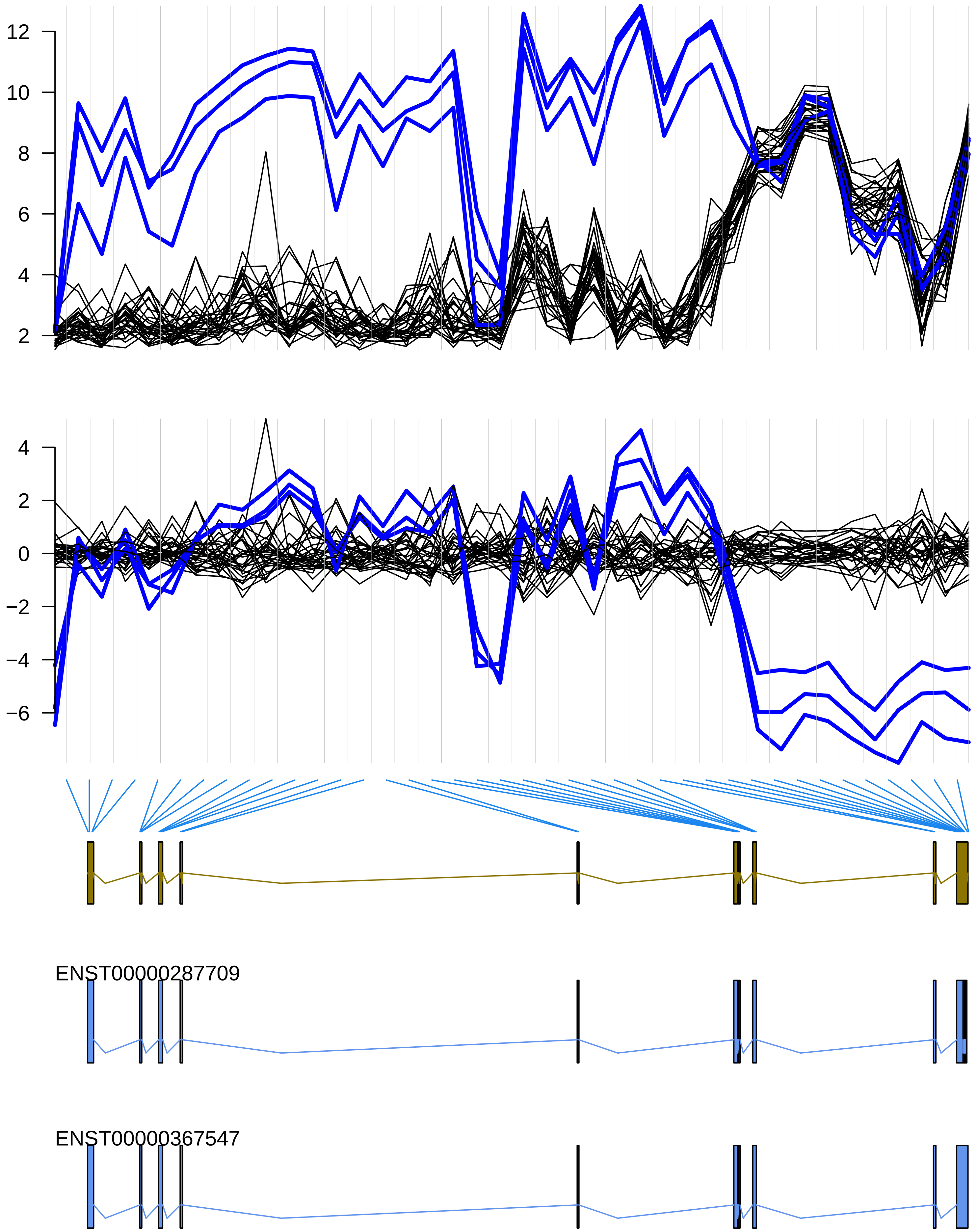

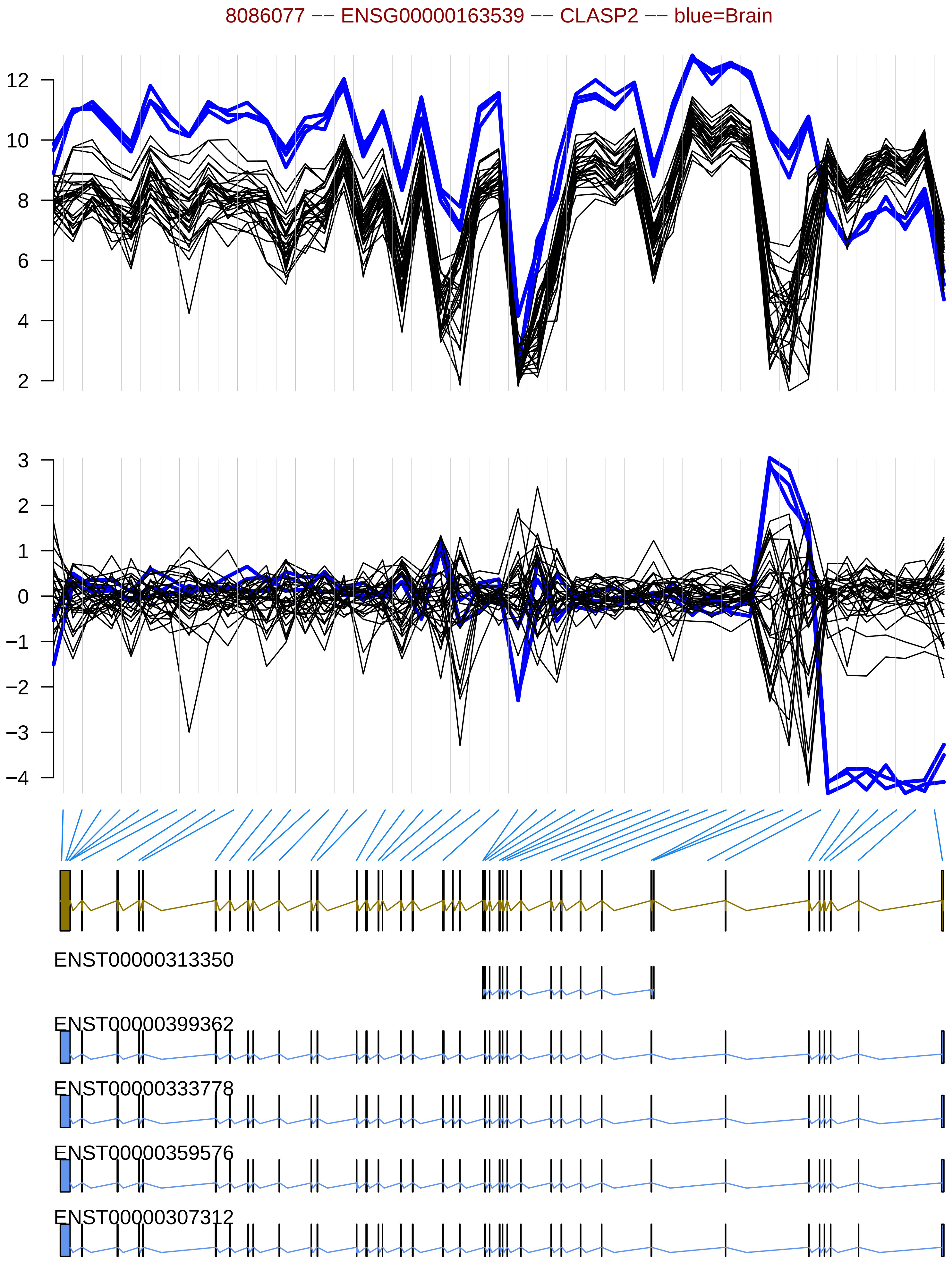
8086077 -- ENSG00000163539 -- CLASP2 -- blue=Brain
12
10
8
6
4
2
3
2
1
0
−1
−2
−3
−4
ENST00000313350
ENST00000399362
ENST00000333778
ENST00000359576
ENST00000307312

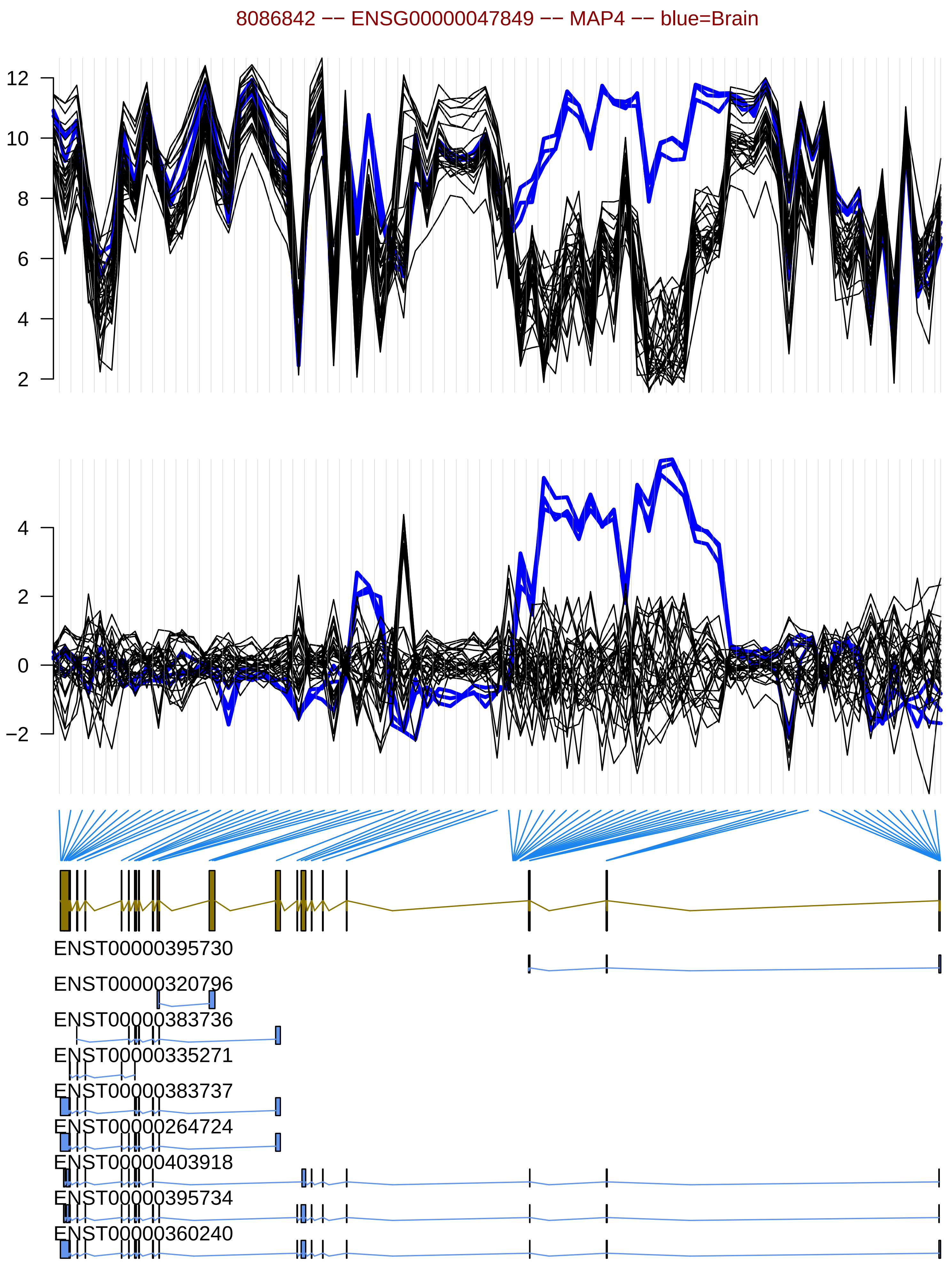
8086842 -- ENSG00000047849 -- MAP4 -- blue=Brain
12
10
8
6
4
2
4
2
0
−2
ENST00000395730
ENST00000320796
ENST00000383736
ENST00000335271
ENST00000383737
ENST00000264724
ENST00000403918
ENST00000395734
ENST00000360240

7957746 -- ENSG00000075415 -- SLC25A3 -- blue=SkMus

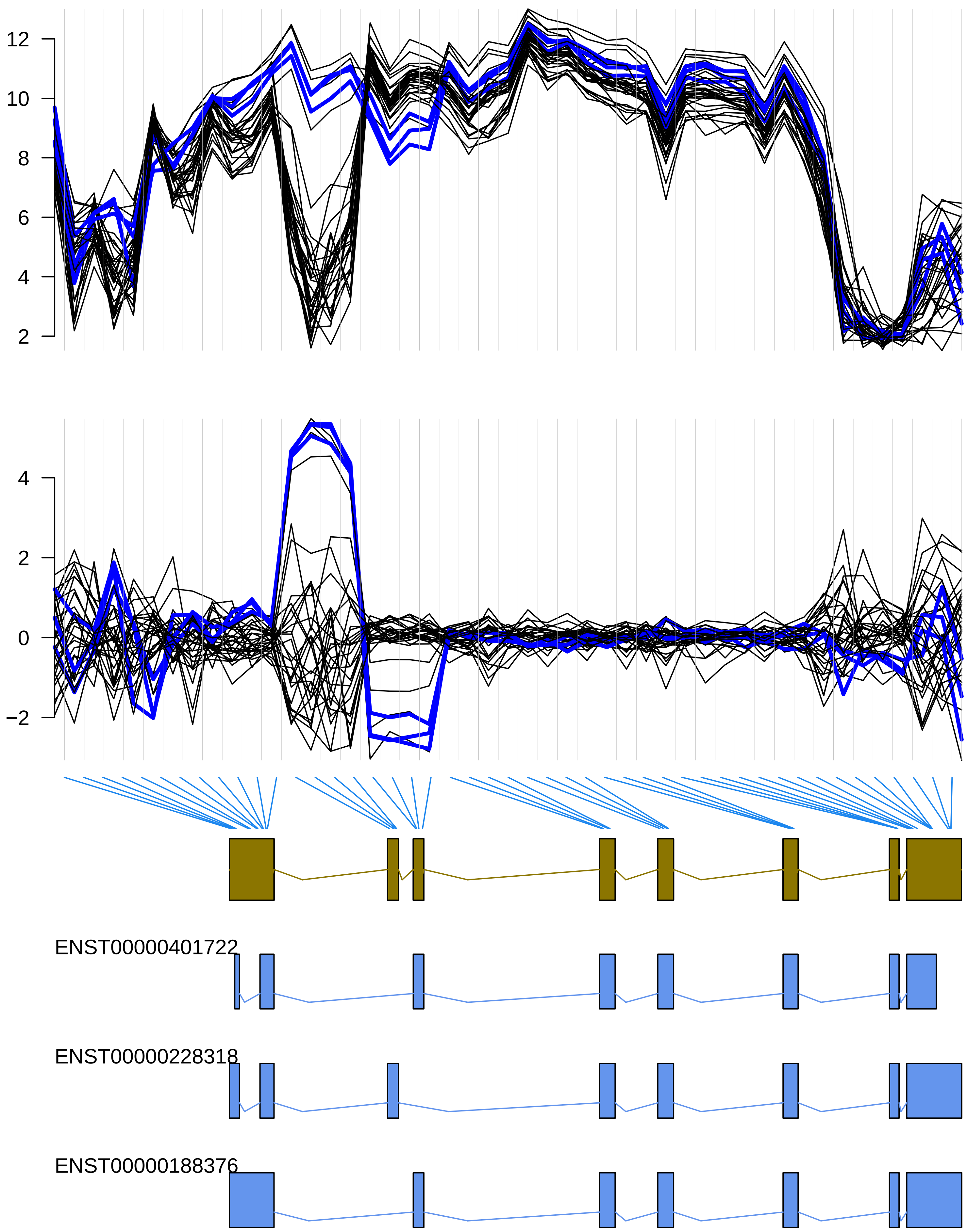

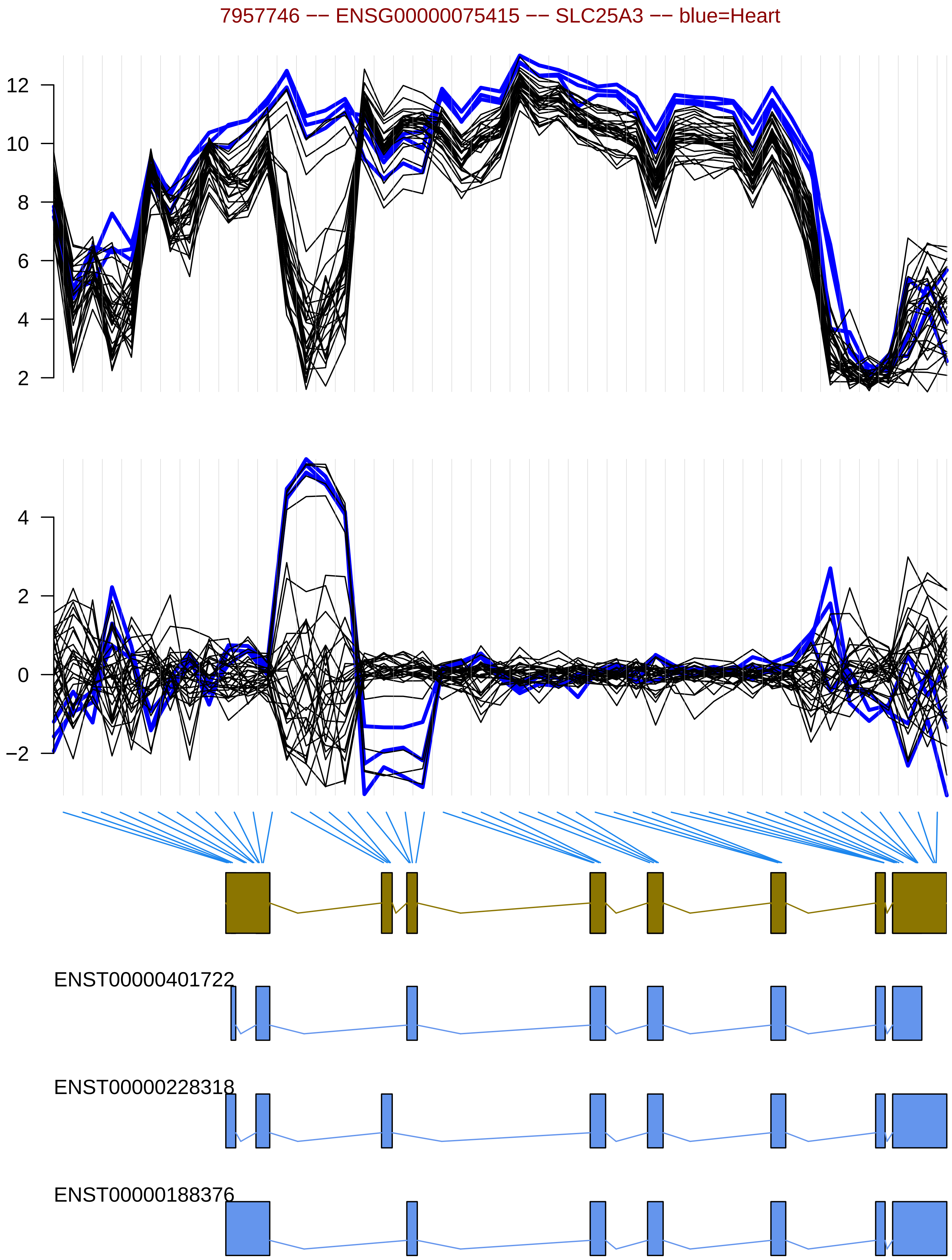

7957746 -- ENSG00000075415 -- SLC25A3 -- blue=Heart
12
10
8
6
4
2
4
2
0
−2
ENST00000401722
ENST00000228318
ENST00000188376

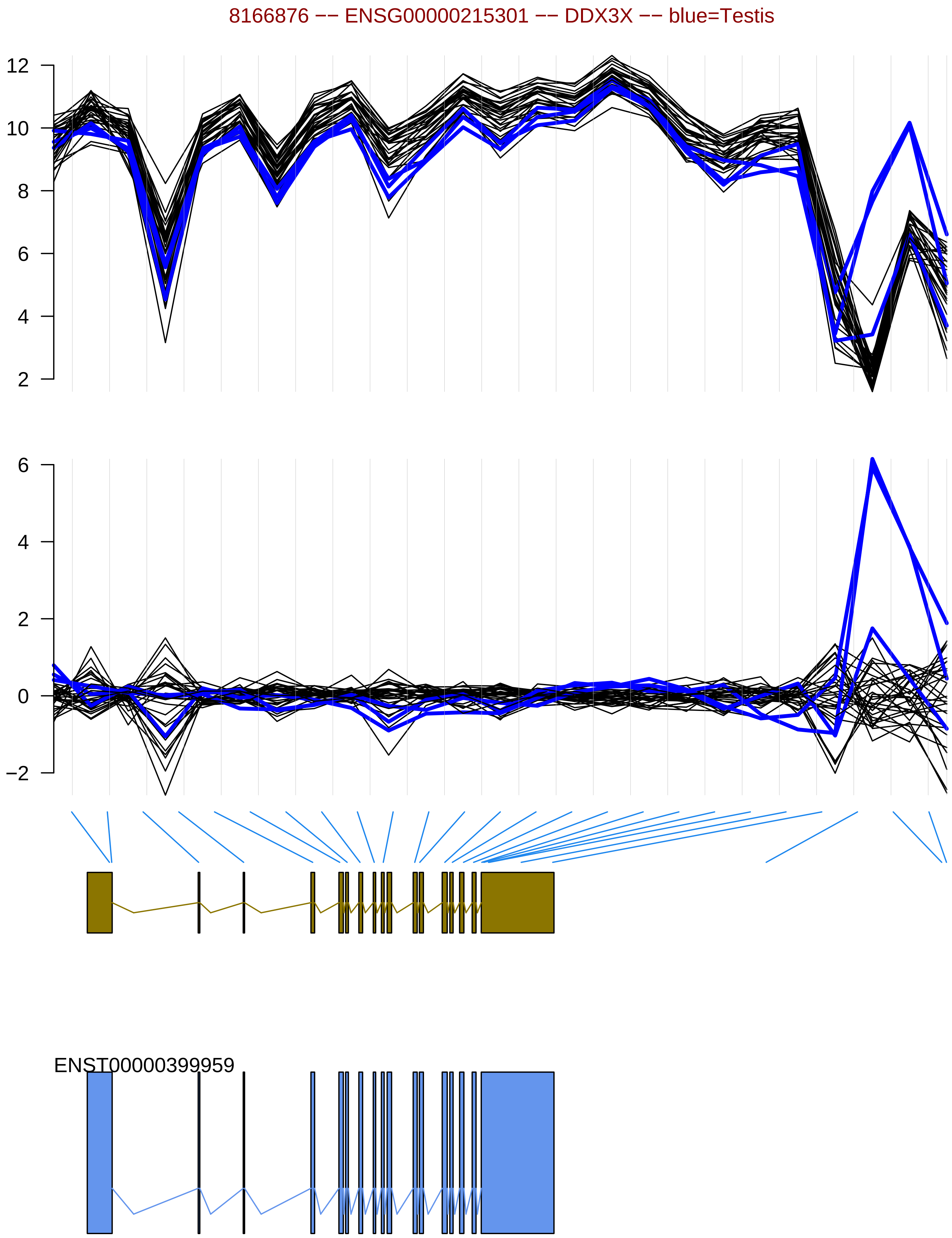
8166876 -- ENSG00000215301 -- DDX3X -- blue=Testis
12
10
8
6
4
2
6
4
2
0
−2
ENST00000399959

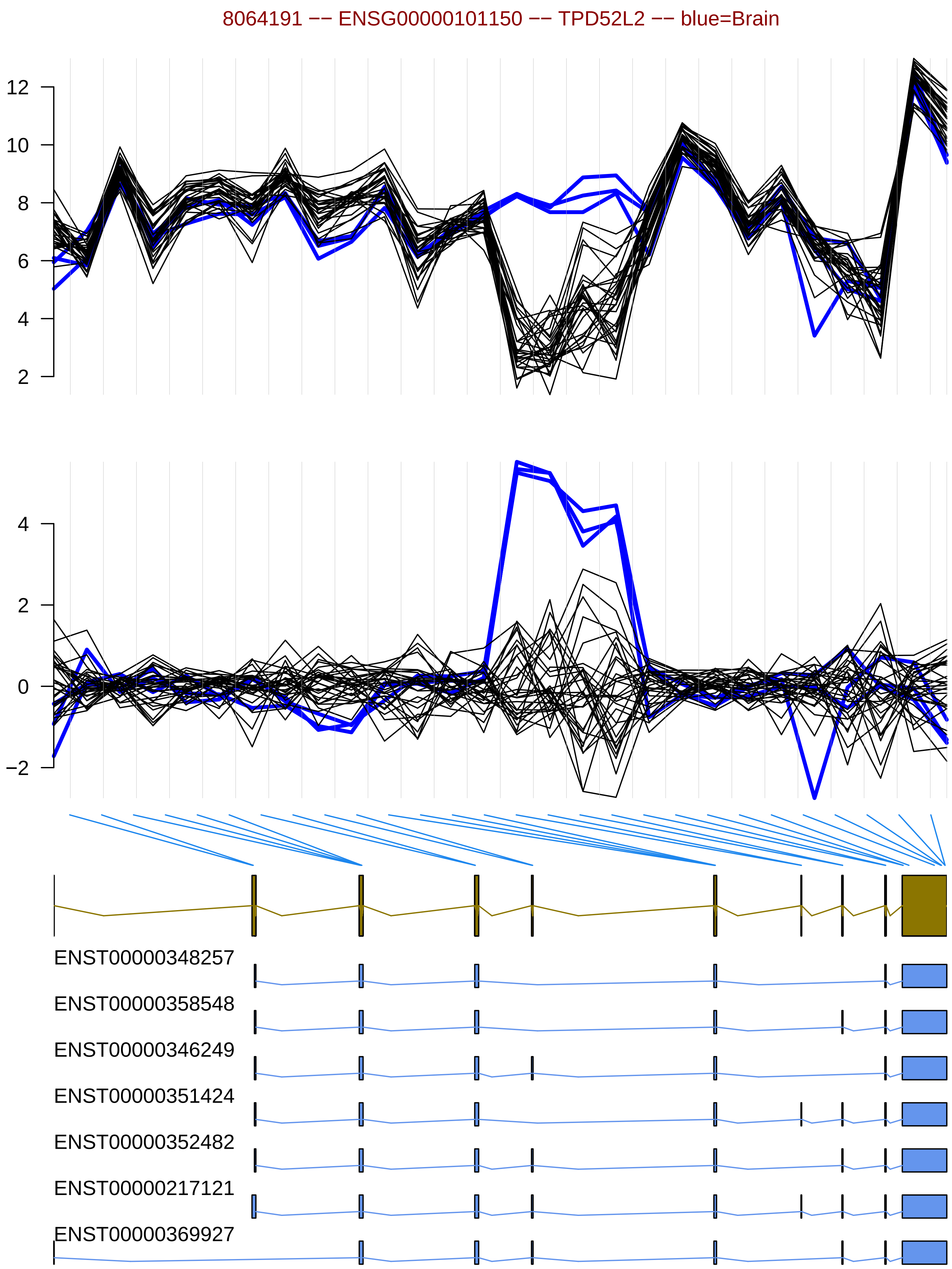
8064191 –– ENSG00000101150 –– TPD52L2 –– blue=Brain
12
10
8
6
4
2
4
2
0
−2
ENST00000348257
ENST00000358548
ENST00000346249
ENST00000351424
ENST00000352482
ENST00000217121
ENST00000369927

8007188 −− ENSG00000173786 −− CNP −− blue=Brain

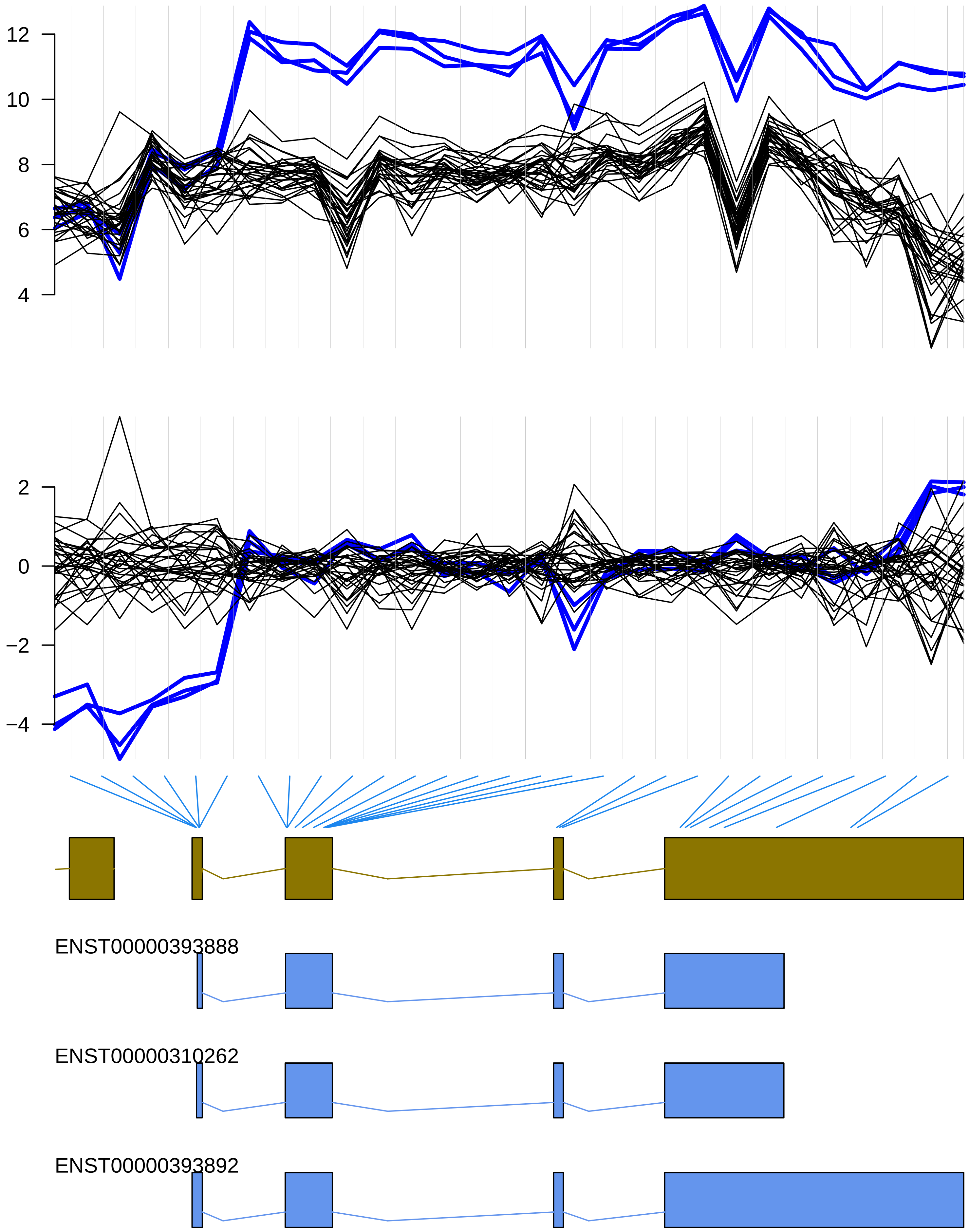

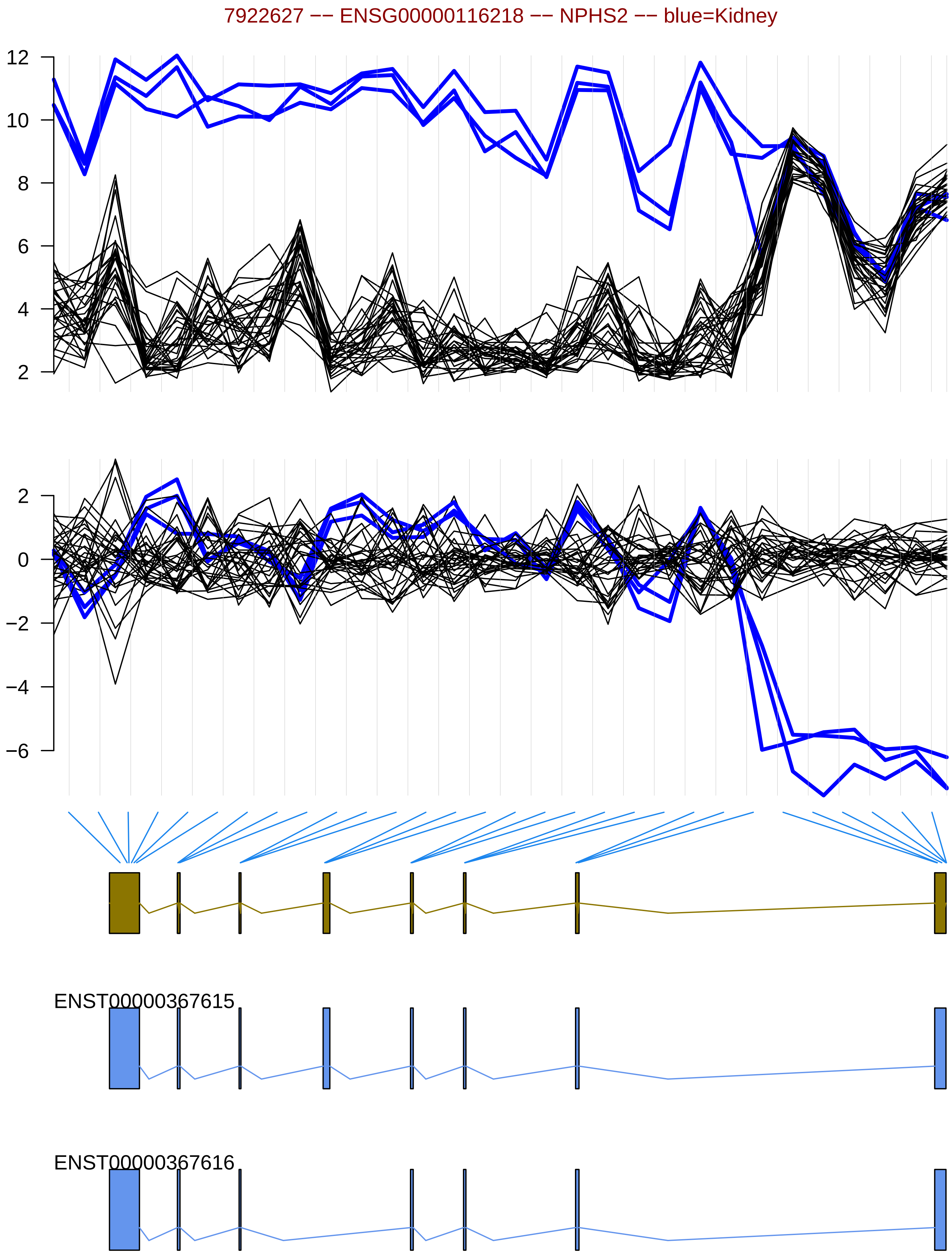
7922627 –– ENSG00000116218 –– NPHS2 –– blue=Kidney
12
10
8
6
4
2
2
0
−2
−4
−6
ENST00000367615
ENST00000367616

8170215 -- ENSG00000101981 -- F9 -- blue=Liver

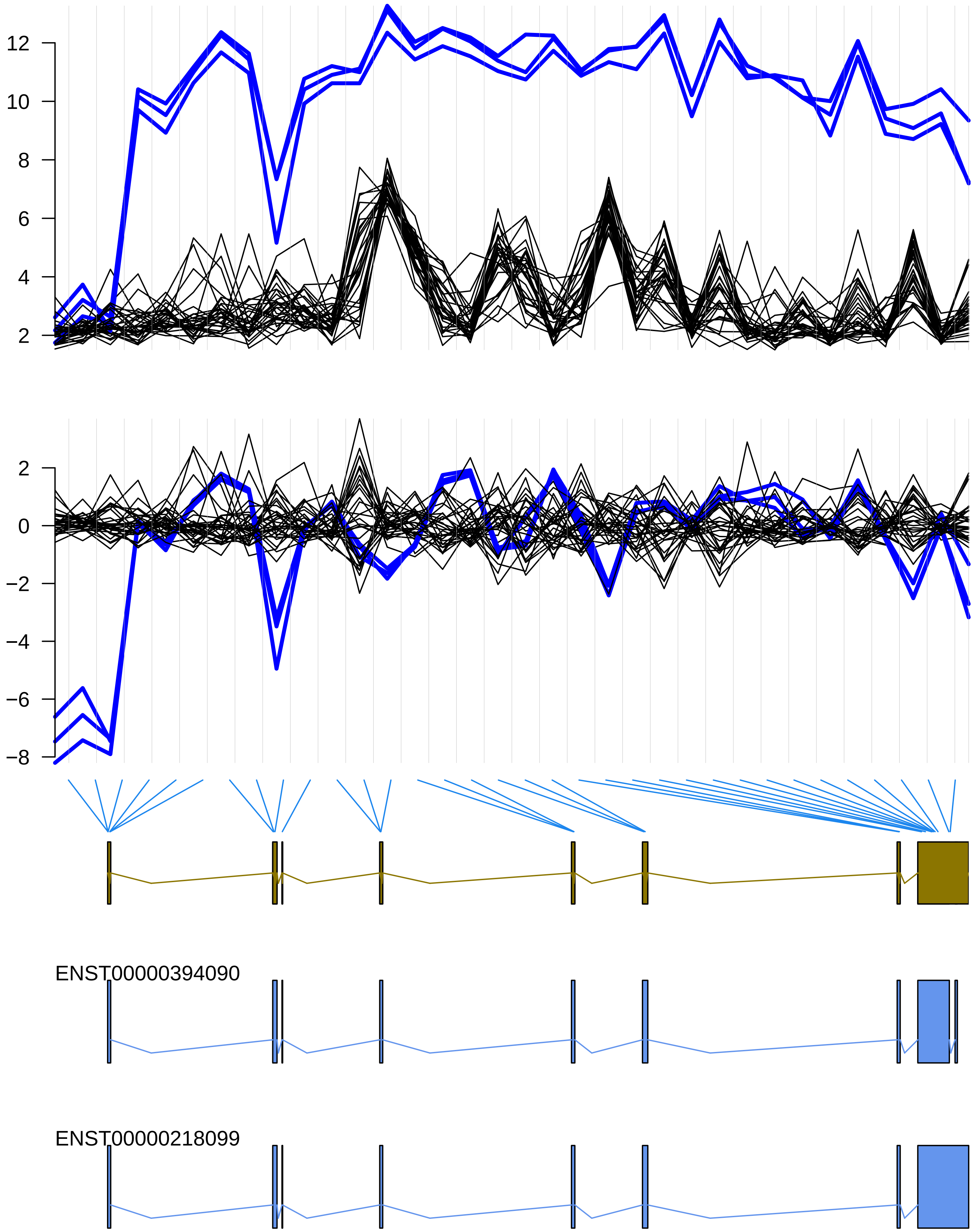

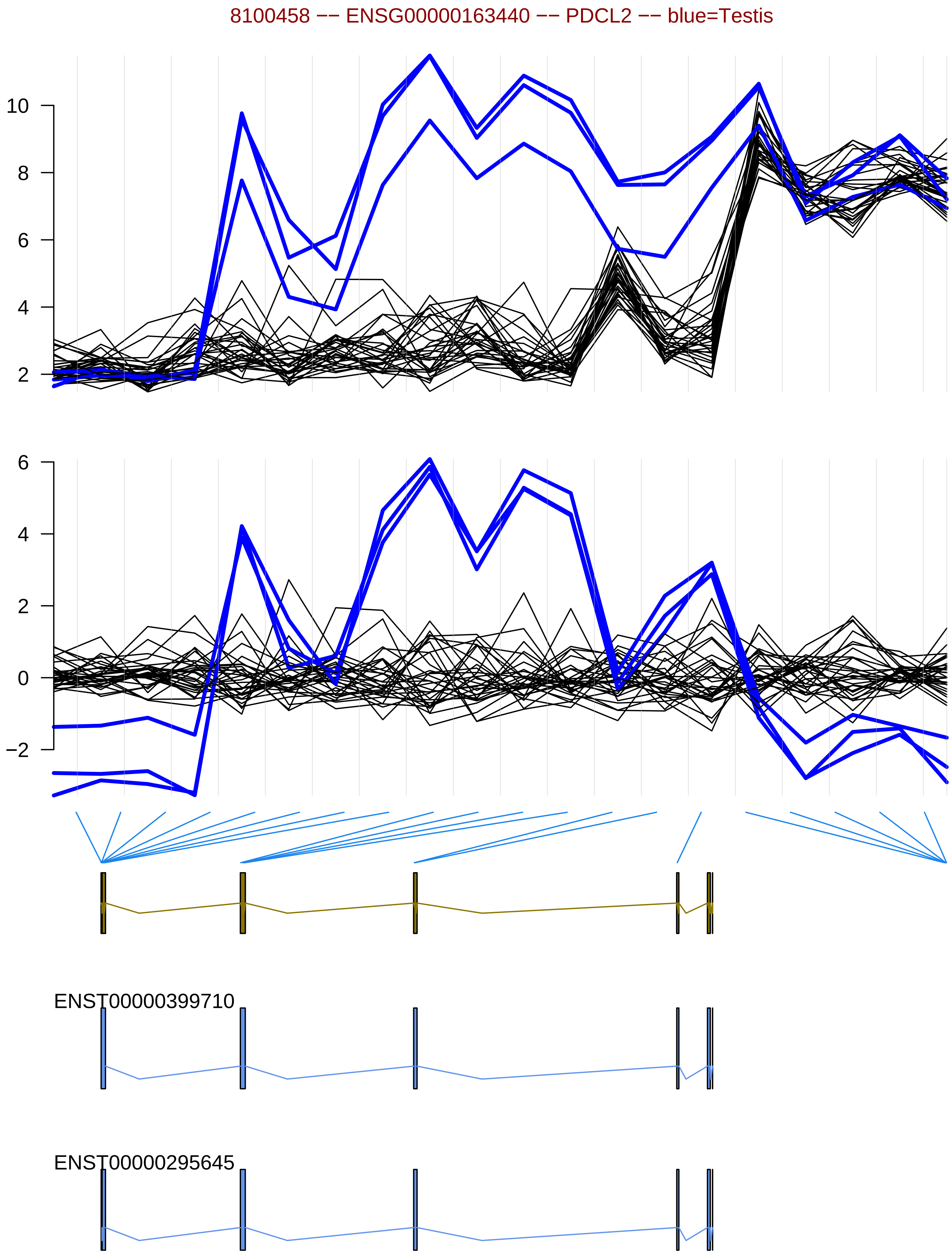
8100458 –– ENSG00000163440 –– PDCL2 –– blue=Testis
10
8
6
4
2
6
4
2
0
−2
ENST00000399710
ENST00000295645

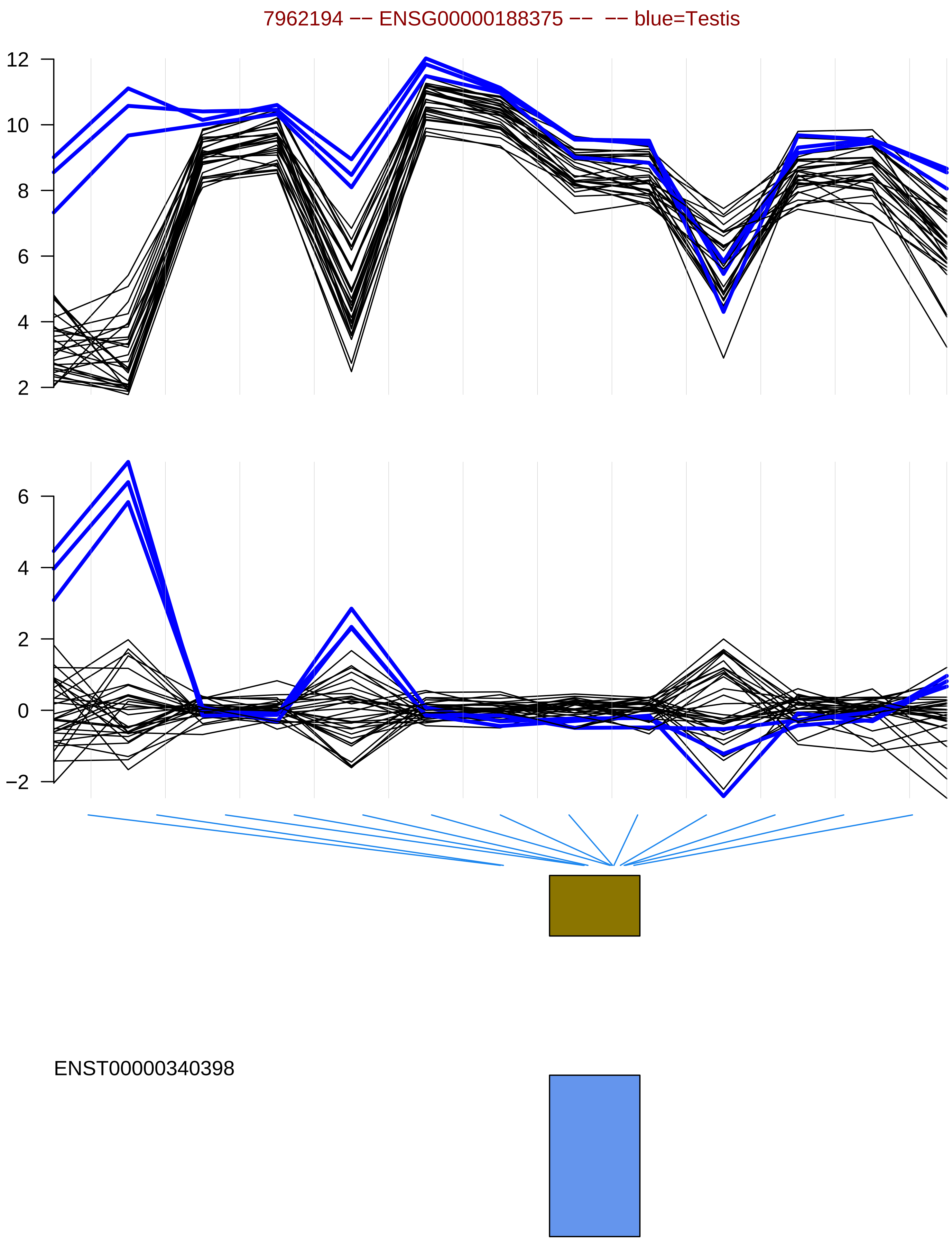

7962194 −− ENSG00000188375 −− −− blue=Testis
12
10
8
6
4
2
6
4
2
0
−2
ENST00000340398

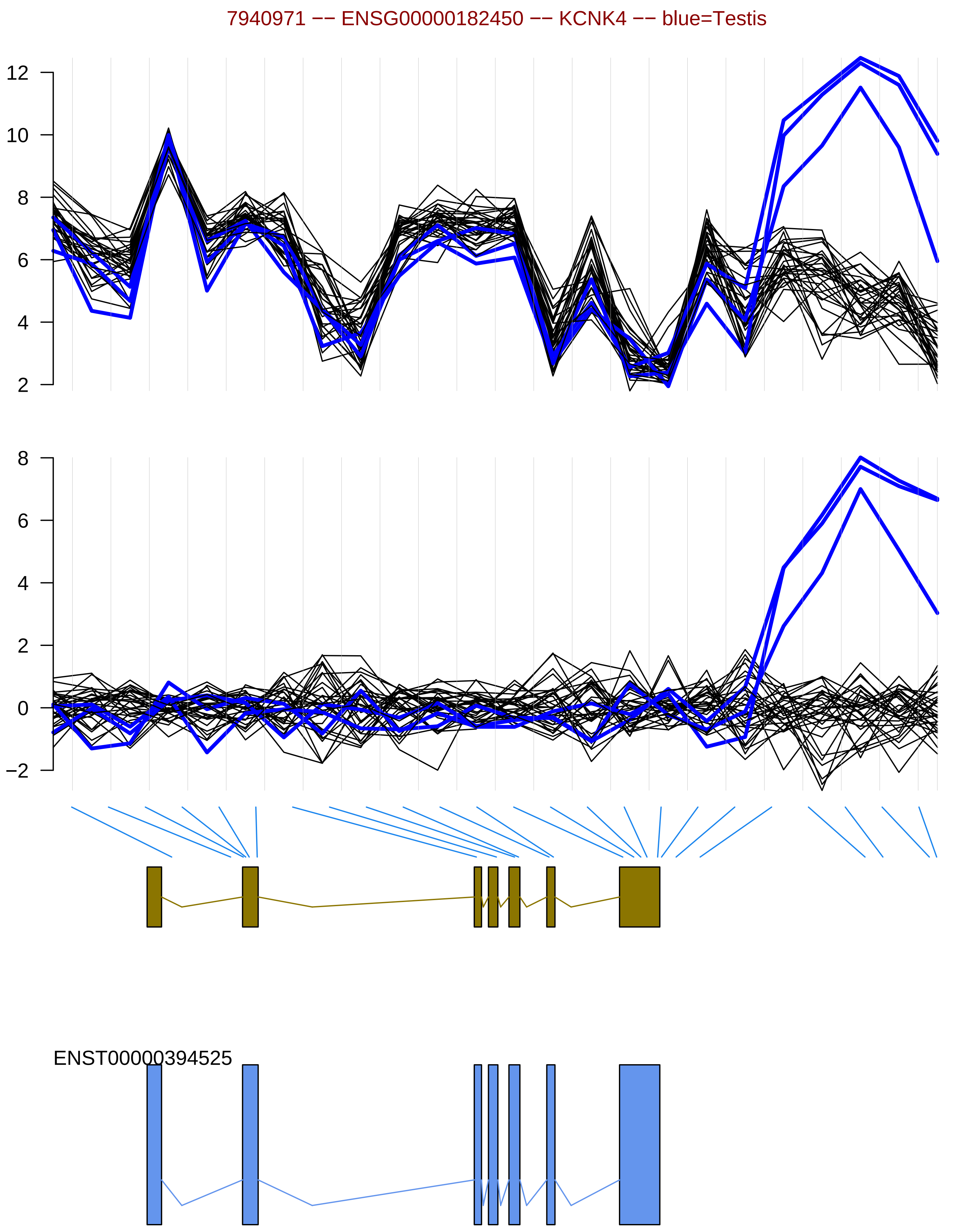
7940971 -- ENSG00000182450 -- KCNK4 -- blue=Testis
12
10
8
6
4
2
8
6
4
2
0
−2
ENST00000394525

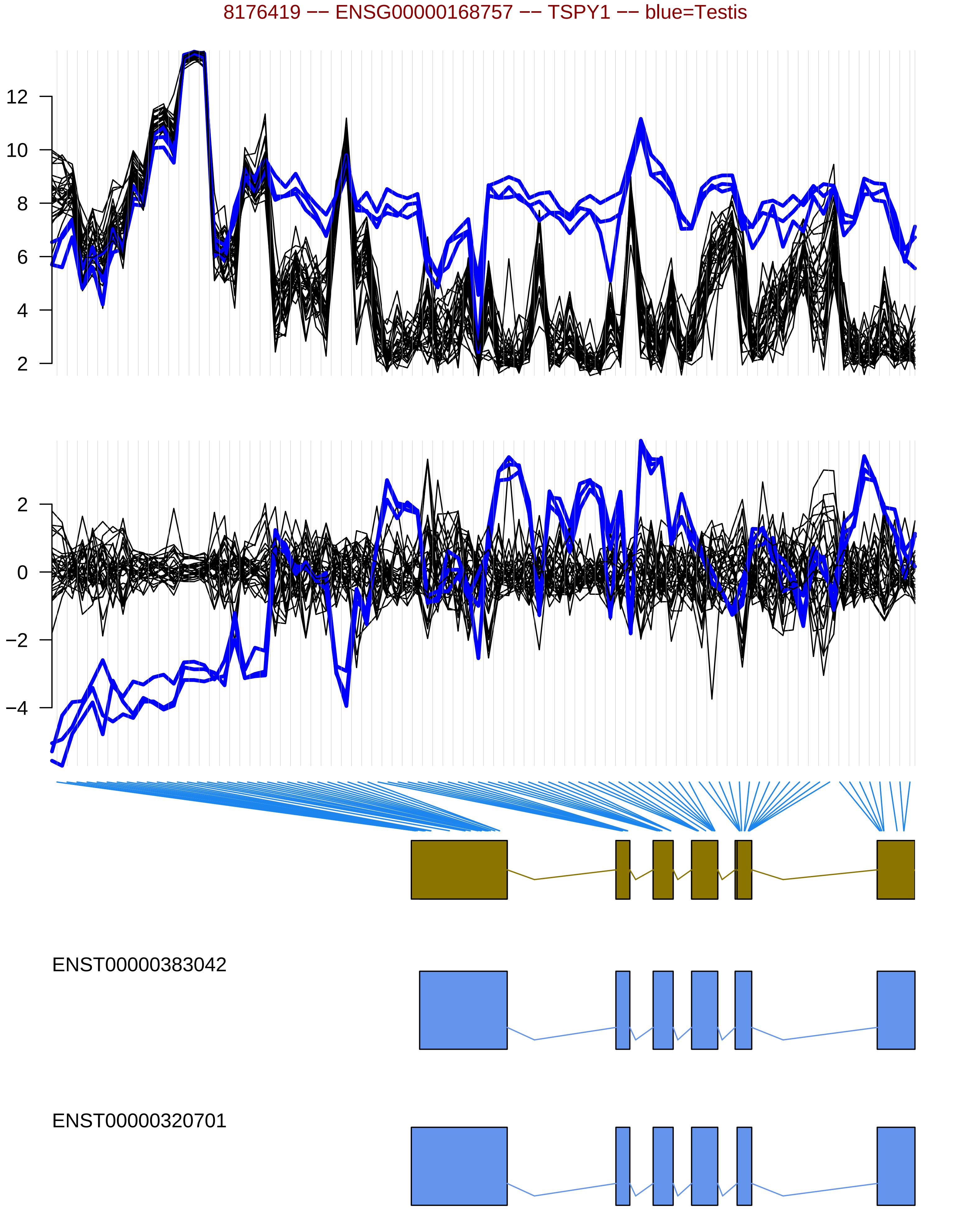

8176419 –– ENSG00000168757 –– TSPY1 –– blue=Testis
12
10
8
6
4
2
2
0
−2
−4
ENST00000383042
ENST00000320701

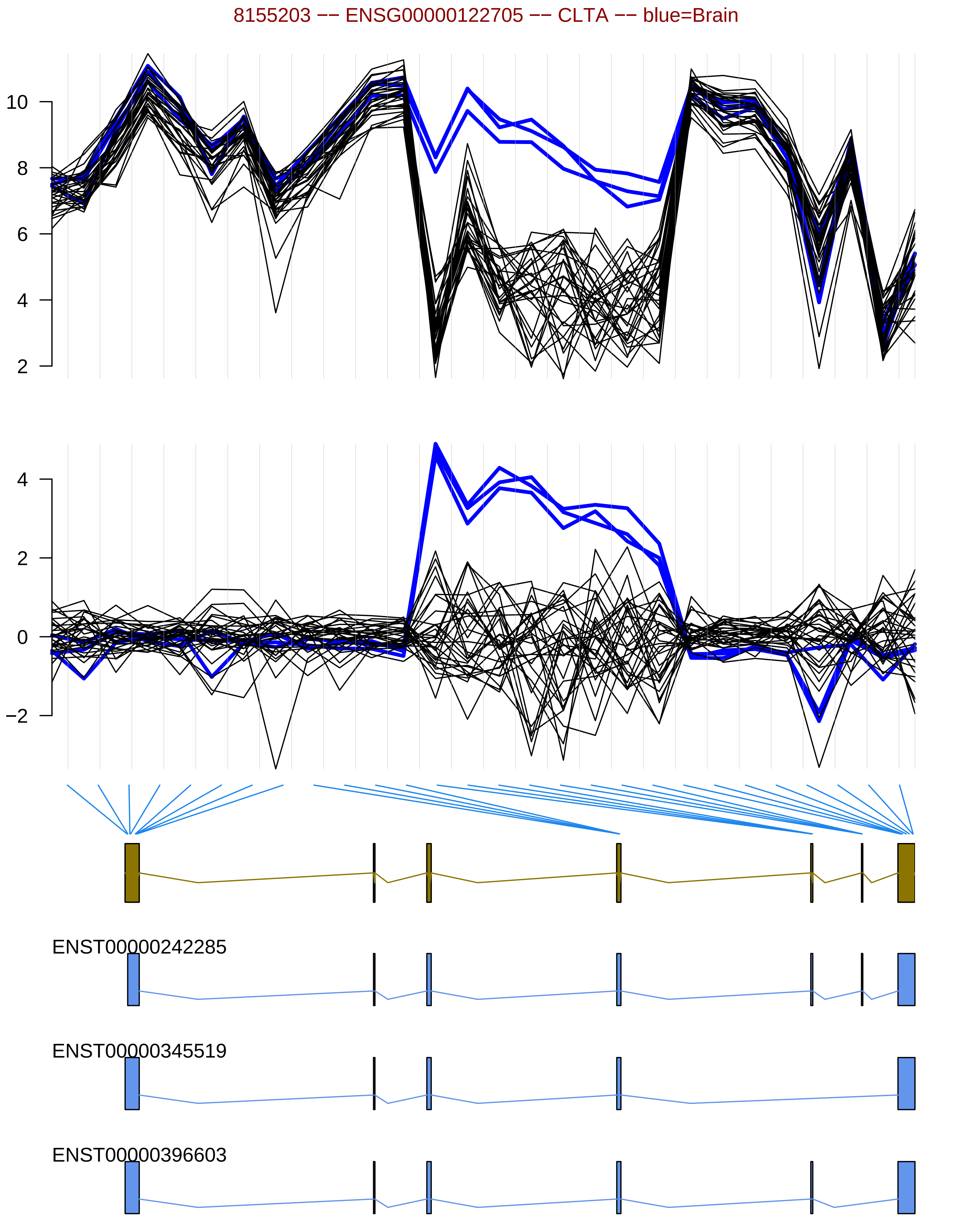
8155203 -- ENSG00000122705 -- CLTA -- blue=Brain
10
8
6
4
2
4
2
0
−2
ENST00000242285
ENST00000345519
ENST00000396603

8170390 −− ENSG00000176289 −− −− blue=Brain

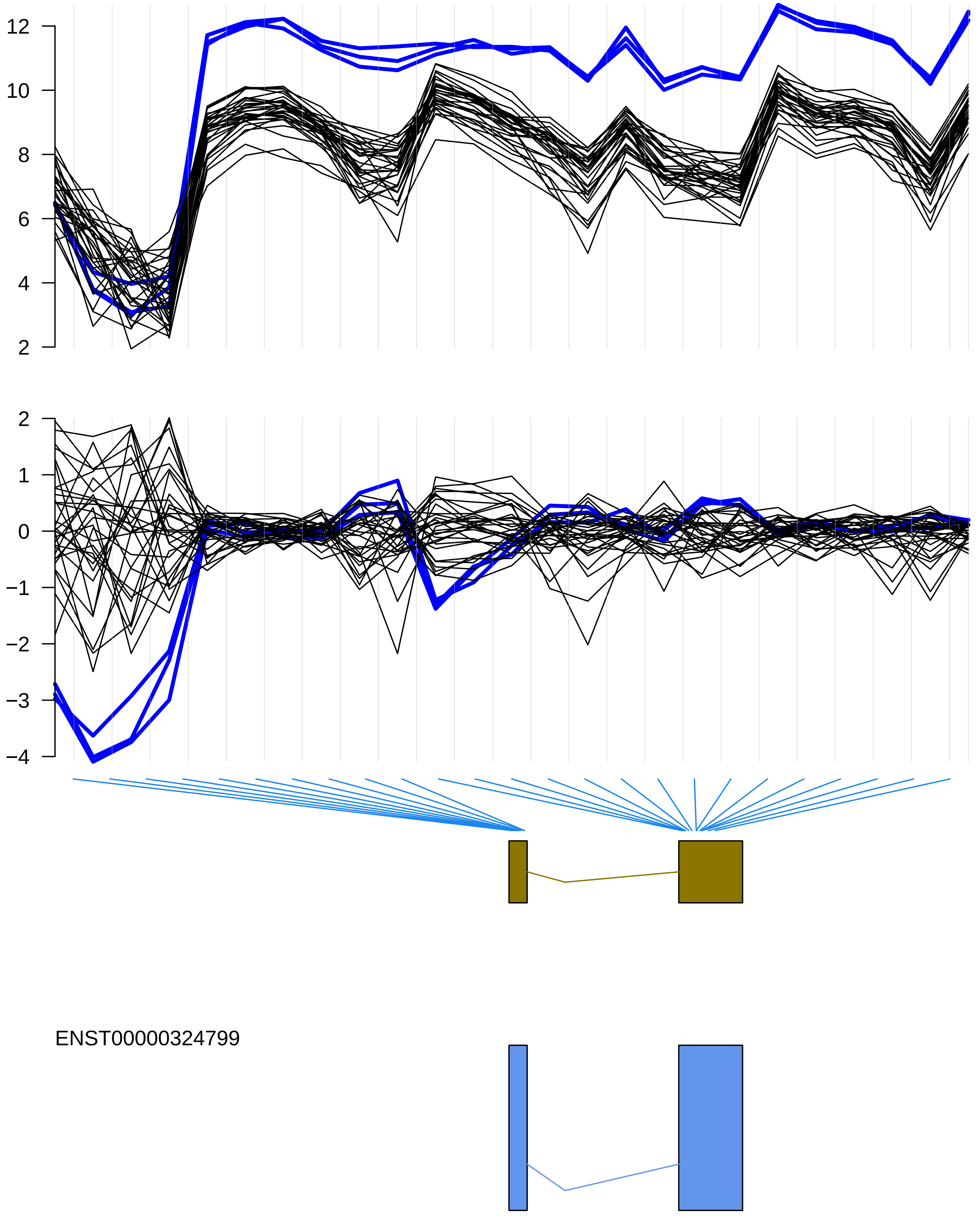

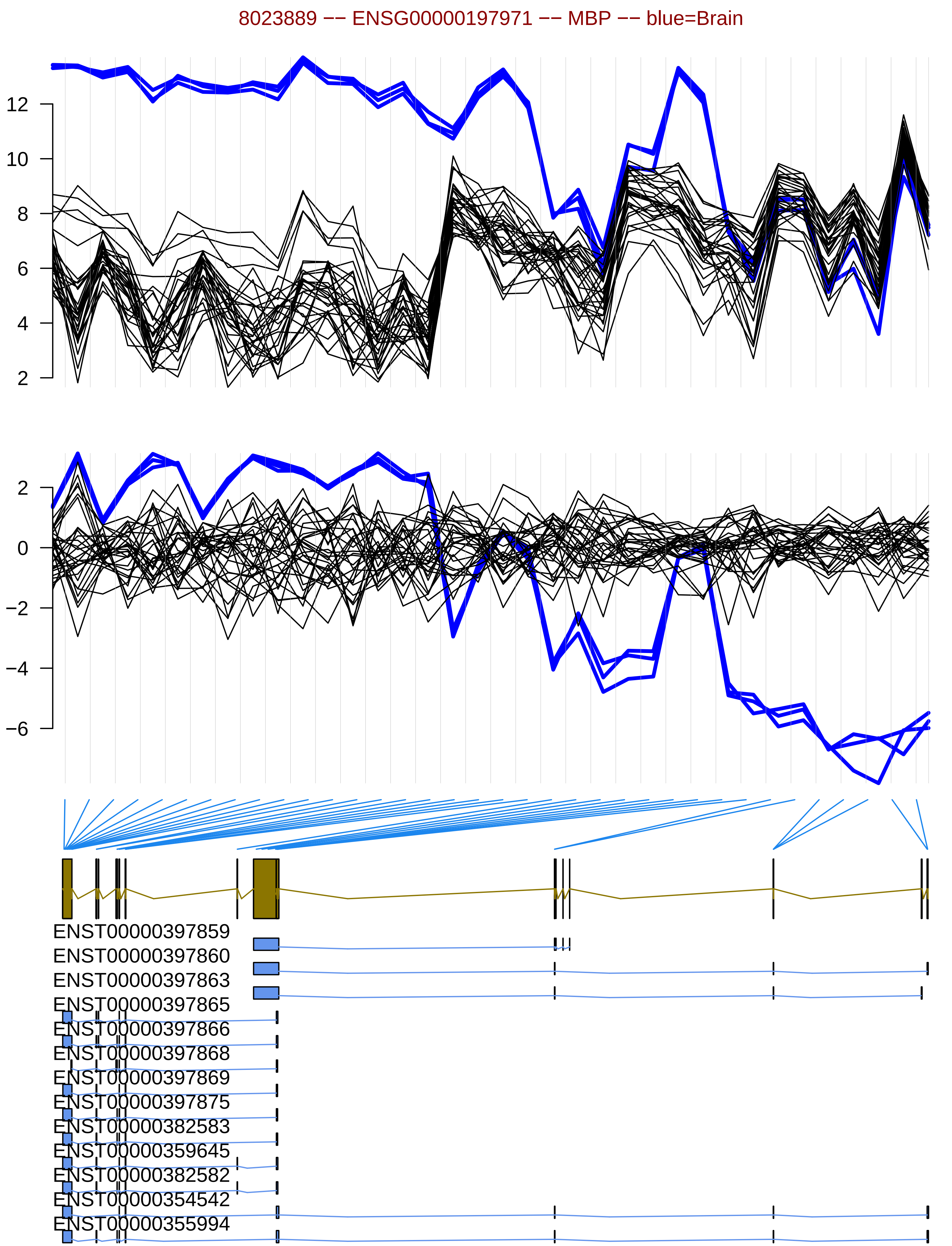
8023889 -- ENSG00000197971 -- MBP -- blue=Brain
12
10
8
6
4
2
2
0
−2
−4
−6
ENST00000397859
ENST00000397860
ENST00000397863
ENST00000397865
ENST00000397866
ENST00000397868
ENST00000397869
ENST00000397875
ENST00000382583
ENST00000359645
ENST00000382582
ENST00000354542
ENST00000355994

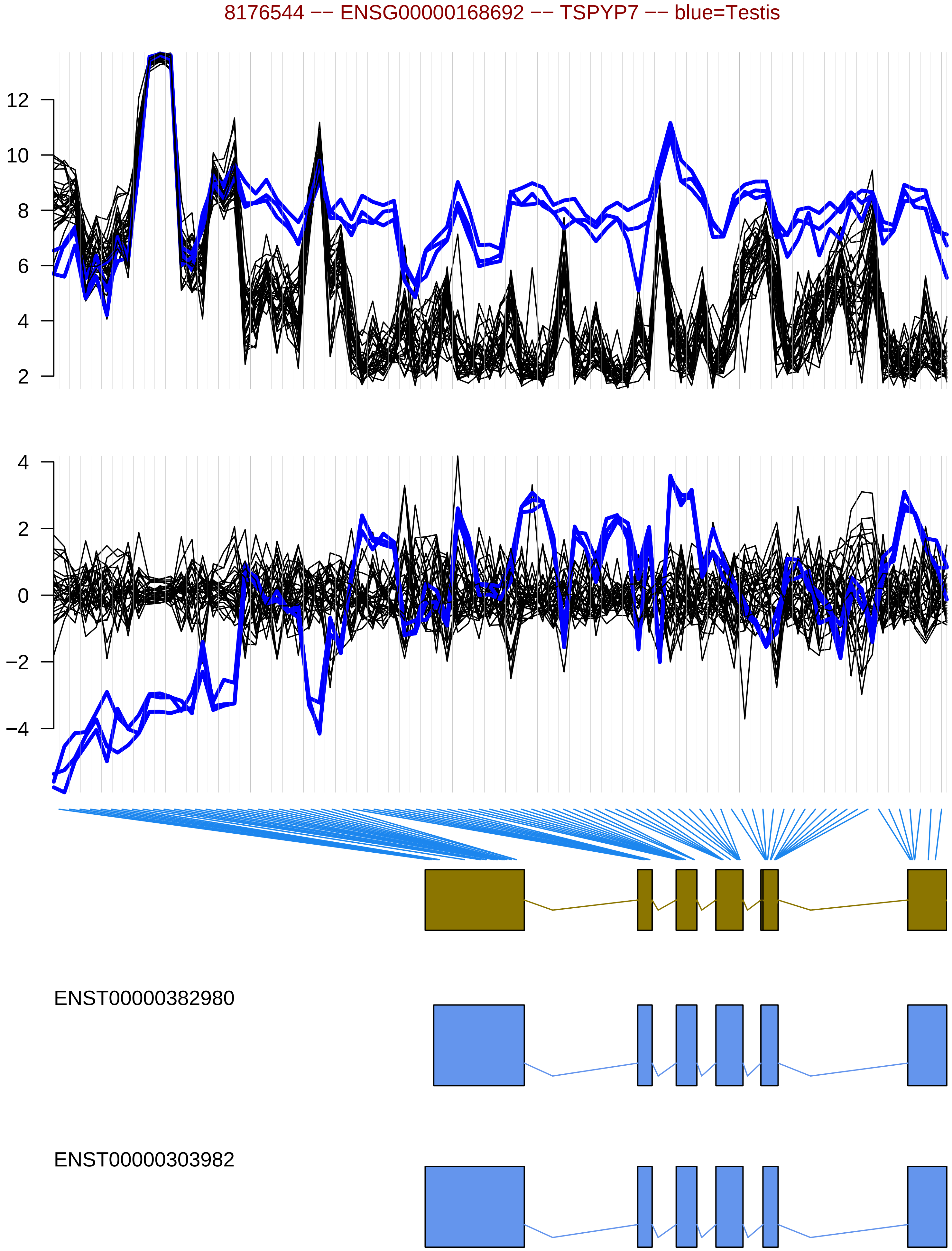

8176544 −− ENSG00000168692 −− TSPYP7 −− blue=Testis
12
10
8
6
4
2
4
2
0
−2
−4
ENST00000382980
ENST00000303982

8024194 -- ENSG00000167468 -- GPX4 -- blue=Testis

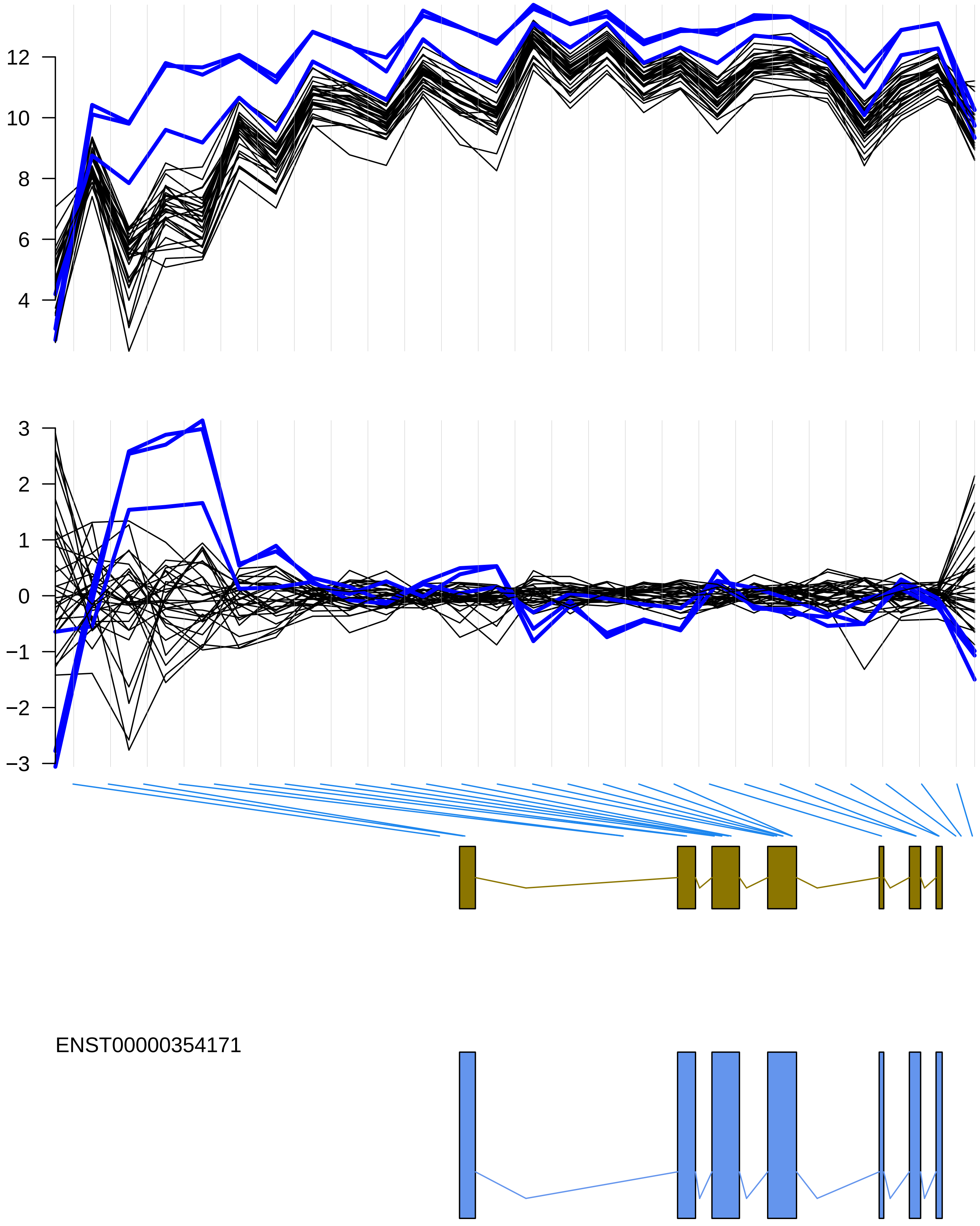

C1orf14

Evidence:

UniProt/Swiss-Prot: CA014_HUMAN, Q9BZQ2
Tissue specificity: Highly expressed in the testis

Reference:

http://www.genecards.org/cgi-bin/carddisp.pl?gene=C1orf14

CLASP2

Evidence:

“These two isoforms probably arise from differential splicing (Espinosa & Navarro 1998). The larger, 170 kDa {alpha}-isoform of CLASP2 (Akhmanova et al. 2001) was detected by western blot in all stages from GV oocytes to two-cell embryos, the brain extract contained the lower weight ß-isoform in addition, as previously reported (Akhmanova et al. 2001).”

Reference:

Reproduction (2005) 130 311-320

PAR-1 and the microtubule-associated proteins CLASP2 and dynactin-p50 have specific localisation on mouse meiotic and first mitotic spindles

Catherine A Moore and Magdalena Zernicka-Goetz

MAP4

Evidence:

Expression of multiple MAP4 isoforms from this gene, which appears to result from alternative RNA splicing, was investigated by RNase protection analysis of mammalian cell lines and rat tissues. The five-repeat isoform was the only form detectable in most cell lines, and it was the most abundant isoform expressed in rat lung, liver, kidney, spleen, and testis. However, in rat brain, heart, and skeletal muscle, although the five-repeat isoform was expressed at all developmental stages examined, the tau-like four-repeat isoform was also expressed, and its expression increased during development. The three-repeat isoform was expressed in heart and, to a lesser extent, in brain, skeletal muscle, and lung"

Reference:

Biochemistry. 1995 34(7):2289-301

Differential expression of alternatively spliced forms of MAP4: a repertoire of structurally different microtubule-binding domains.

Chapin SJ, Lue CM, Yu MT, Bulinski JC.

SLC25A3

Evidence:

"These two isoforms originate from alternatively spliced transcripts that differ in a mutually exclusive exon; exon 3A has tissue-specific expression in heart and muscle, whereas exon 3B is the predominant isoform in other tissues."

Reference:

Am J Hum Genet. 2007 March; 80(3): 478–484.

Mitochondrial Phosphate–Carrier Deficiency: A Novel Disorder of Oxidative Phosphorylation

Johannes A. Mayr, Olaf Merkel,* Sepp D. Kohlwein, Boris R. Gebhardt, Hansjosef Böhles, Ulrike Fötschl, Johannes Koch, Michaela Jaksch,† Hanns Lochmüller, Rita Horváth, Peter Freisinger, and Wolfgang Sperl

DDX3X

Evidence:

“While DDX3Y, which is essential for normal spermatogenesis, is translated only in the testes, DDX3X protein is ubiquitously expressed, involved in RNA transcription, RNA splicing, mRNA transport, translation initiation and cell cycle regulation.”

Reference:

Curr Med Chem. 2007 14(23):2517-25.
The DDX3 subfamily of the DEAD box helicases: divergent roles as unveiled by studying different organisms and in vitro assays.

Rosner A, Rinkevich B.

TPD52L2

Evidence:

“In total, we confirmed 14 genes with glioma-specific splicing; seven were novel events identified by the exon expression array (A2BP1, BCAS1, CACNA1G, CLTA, KCNC2, SNCB, and TPD52L2).”

Reference:

BMC Genomics. 2008 May 12;9:216

Global analysis of aberrant pre-mRNA splicing in glioblastoma using exon expression arrays.
Cheung HC, Baggerly KA, Tsavachidis S, Bachinski LL, Neubauer VL, Nixon TJ, Aldape KD, Cote GJ, Krahe R.

Also:
http://splicenest.molgen.mpg.de/Tissue_Tumor_Specificity/Tumor.html

CNP

Evidence:

“Analysis of the expression of the CNP (2prime3prime cyclic nucleotide-3prime-phosphodiesterase) gene indicated that in both cell lines only one of the two CNP isoforms was expressed at 34°C.”

Reference:

Temperature-Dependent Regulation of PLP/DM20 and CNP Gene Expression in Two Conditionally-Immortalized Jimpy Oligodendrocyte Cell Lines
Neurochemical Research
Volume 22, Number 4 / April, 1997
E. R. Bongarzone, L. M. Foster, S. Byravan, V. Schonmann and A. T. Campagnoni

NPHS2

Evidence:

“We recently cloned a novel gene, NPHS2, involved in autosomal recessive steroid-resistant nephrotic syndrome. This gene encodes a novel podocyte protein, podocin … In the mature kidney, NPHS2 is exclusively expressed in the podocytes of mature glomeruli.”

Reference:

Am J Pathol. 2002 January; 160(1): 131–139.

Podocin Localizes in the Kidney to the Slit Diaphragm Area

Séverine Roselli,* Olivier Gribouval,* Nicolas Boute,* Mireille Sich,* France Benessy,* Tania Attié,† Marie-Claire Gubler,* and Corinne Antignac*

F9

Evidence:

Liver-Specific Expression, supplementary materials.

Reference:

Thrombosis and Haemostasis 2002; 87: 366-373

Expression of Coagulation Factor IX in a Haematopoietic Cell Line*

M. H. Rodriguez, N. Enjolras, J. L. Plantier, M. Réa, M. Leboeuf, G. Uzan, J. C. Bordet, C. Négrier

http://82.139.217.185/schatt/zs/thromb/2002/3/pdf/02030366.pdf

PDCL2

Evidence:

"…Expression of the sense and the antisense transcript of Pdcl2 was testis specific…"

Reference:

The complexity of antisense transcription revealed by the study of developing male germ cells

Genomics
Volume 87, Issue 6, June 2006, Pages 681-692

Wai-Yee Chana, Shao-Ming Wua, Lisa Ruszczyka, Evelyn Lawa, Tin-Lap Leea, Vanessa Baxendalea, Alan Lap-Yin Panga and Owen M. Rennerta

LOC440093

Evidence:

None (hypothetical protein LOC440093)

Reference:

KCNK4

Evidence:

The human KCNK4 gene encodes several transcripts that generate two-pore K+ channel subunits … Human KCNK4 transcripts were expressed mainly in the heart and brain but also in the liver, skeletal muscle, kidney and pancreas. In rat, the transcripts were strongly expressed in the brain but were also detected in the lung, kidney, liver, spleen, skeletal muscle, testes and at lower levels in the heart. Expression of HKT4.1b in Xenopus oocytes drives the resting potential close to the potassium equilibrium voltage.

Reference:

Cloning of two transcripts, HKT4.1a and HKT4.1b, from the human two-pore K+ channel gene KCNK4. Chromosomal localization, tissue distribution and functional expression.

Ozaita A, Vega-Saenz de Miera E.
Brain Res Mol Brain Res. 2002 Jun 15;102(1-2):18-27.

TSPY2

Evidence:

Gene name: testis specific protein, Y-linked 2

Reference:

http://www.ncbi.nlm.nih.gov/sites/entrez?Db=gene&TermToSearch=64591

CLTA

Evidence:

3 isoforms: 1 brain, 1 non-brain, 1 other

Reference:

http://www.uniprot.org/uniprot/P09496

MBP

Evidence:

The primer set used for assaying MBP mRNAs consists of the 5' primer in exon 3 and the 3' primer in exon 7. The MBP splicing patterns have been well described (33). All known MBP splice variants include exons 3 and 7.

Reference:

Endocrinology Vol. 145, No. 11 5013-5020

Thyroid Hormone Regulates Oligodendrocyte Accumulation in Developing Rat Brain White Matter Tracts

Christopher M. Schoonover, Melissa M. Seibel, Dawn M. Jolson, Mary Jo Stack, Rounak J. Rahman, Sidney A. Jones, Cary N. Mariash and Grant W. Anderson

Cell 43:721–727 (1985)

Alternative splicing accounts for the four forms of myelin basic protein.

de Ferra F, Engh H, Hudson L, Kamholz J, Puckett C, Molineaux S, Lazzarini RA

TSPY1

Evidence:

Gene name: testis specific protein, Y-linked 1

Reference:

http://www.binfo.ncku.edu.tw/cgi-bin/gf.pl?genename=TSPY1

GPX4

Evidence:

Phospholipid hydroperoxide glutathione peroxidase (GPx4) is a selenocysteine-containing enzyme, and three different isoforms (cytosolic, mitochondrial, and nuclear) originate from the GPx4 gene … High concentrations of GPx4 were found in testis …

Reference:

J. Biol. Chem., Vol. 281, Issue 28, 19655-19664, July 14, 2006

The Role of Phospholipid Hydroperoxide Glutathione Peroxidase Isoforms in Murine Embryogenesis*

Astrid Borchert, Chi Chiu Wang, Christoph Ufer, Heike Schiebel, Nicolai E. Savaskan, and Hartmut Kuhn
